# Spliceosomal profiling identifies EIF4A3 as a novel oncogene in hepatocellular carcinoma acting through the modulation of FGFR4 splicing

**DOI:** 10.1002/ctm2.1102

**Published:** 2022-11-23

**Authors:** Juan L. López‐Cánovas, Natalia Hermán‐Sánchez, Maria Trinidad Moreno‐Montilla, Mercedes del Rio‐Moreno, Emilia Alors‐Perez, Marina E. Sánchez‐Frias, Víctor Amado, Rubén Ciria, Javier Briceño, Manuel de la Mata, Justo P. Castaño, Manuel Rodriguez‐Perálvarez, Raúl M. Luque, Manuel D. Gahete

**Affiliations:** ^1^ Maimonides Biomedical Research Institute of Cordoba (IMIBIC) Cordoba Spain; ^2^ Department of Cell Biology Physiology and Immunology University of Córdoba Cordoba Spain; ^3^ Reina Sofía University Hospital Cordoba Spain; ^4^ CIBER Pathophysiology of Obesity and Nutrition (CIBERobn) Cordoba Spain; ^5^ Department of Hepatology and Liver Transplantation Reina Sofía University Hospital Cordoba Spain; ^6^ CIBER Hepatic and Digestive Diseases (CIBERehd) Cordoba Spain; ^7^ Unit of Hepatobiliary Surgery and Liver Transplantation University Hospital Reina Sofia Cordoba Spain

**Keywords:** FGF19, liver cancer, preclinical model, splicing machinery

## Abstract

**Introduction:**

Altered splicing landscape is an emerging cancer hallmark; however, the dysregulation and implication of the cellular machinery controlling this process (spliceosome components and splicing factors) in hepatocellular carcinoma (HCC) is poorly known. This study aimed to comprehensively characterize the spliceosomal profile and explore its role in HCC.

**Methods:**

Expression levels of 70 selected spliceosome components and splicing factors and clinical implications were evaluated in two retrospective and six in silico HCC cohorts. Functional, molecular and mechanistic studies were implemented in three cell lines (HepG2, Hep3B and SNU‐387) and preclinical Hep3B‐induced xenograft tumours.

**Results:**

Spliceosomal dysregulations were consistently found in retrospective and in silico cohorts. *EIF4A3*, *RBM3*, *ESRP2* and *SRPK1* were the most dysregulated spliceosome elements in HCC. *EIF4A3* expression was associated with decreased survival and greater recurrence. Plasma EIF4A3 levels were significantly elevated in HCC patients. In vitro EIF4A3‐silencing (or pharmacological inhibition) resulted in reduced aggressiveness, and hindered xenograft‐tumours growth in vivo, whereas EIF4A3 overexpression increased tumour aggressiveness. EIF4A3‐silencing altered the expression and splicing of key HCC‐related genes, specially FGFR4. *EIF4A3*‐silencing blocked the cellular response to the natural ligand of FGFR4, FGF19. Functional consequences of *EIF4A3*‐silencing were mediated by FGFR4 splicing as the restoration of non‐spliced FGFR4 full‐length version blunted these effects, and FGFR4 inhibition did not exert further effects in *EIF4A3*‐silenced cells.

**Conclusions:**

Splicing machinery is strongly dysregulated in HCC, providing a source of new diagnostic, prognostic and therapeutic options in HCC. EIF4A3 is consistently elevated in HCC patients and associated with tumour aggressiveness and mortality, through the modulation of FGFR4 splicing.

## Graphical abstract

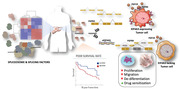

## 1 INTRODUCTION

Hepatocellular carcinoma (HCC) is the sixth cancer type in incidence and represents the most prevalent type of primary liver cancer worldwide.^1^, ^2^ However, the molecular determinants underlying HCC development and progression are still to be fully elucidated. Remarkably, human cancers, including HCC, are characterized by the pathological alteration of the splicing process, an essential cellular mechanism that governs many aspects of cellular proliferation, survival and differentiation and that represents a novel cancer hallmark.^3^ Previous reports indicate that aberrant splicing variants of relevant genes, such as *CDCC50*, *KLF6*, *FN1 or TP73*, are associated with liver carcinogenesis, thus suggesting that an altered splicing process could play an essential role in the development and progression of HCC.^4^, ^5^, ^6^ Consistent with this idea, the splicing profile is altered in earlier stages of the pathological progression of HCC, including fatty liver disease,^7^, ^8^ and specific alternative splicing signatures may predict HCC prognosis, tumour spread and survival.^9^

Perturbations in the splicing process are frequently found in different cancer types associated with mutations and/or alterations in the expression of the components of the cellular machinery that controls the splicing process.^10^ The splicing process is controlled and catalysed by the spliceosome, a dynamic intracellular machinery comprised by several macromolecular complexes of ribonucleoproteins, which includes a central small nuclear RNA (snRNA) and a set of interacting proteins. Spliceosome is subdivided into major or U2‐dependent spliceosome, consisting of five snRNA (U1, U2, U5 and U4/U6), and minor or U12‐dependent spliceosome (U11, U12, U5 and U4atac/U6atac).^11^ This process is finely modulated by more than 300 accessory proteins named splicing factors (e.g. SRSF1, SF3B1 and ESRP1), which are also essential for the appropriate splicing process.^12^

Dysregulations in the spliceosomal landscape (spliceosome components and splicing factors) induce altered and/or aberrant splicing processes, which may be associated with the development and progression of different pathologies, including diabetes,^13^ fatty liver disease^8^ and tumour pathologies (pituitary and pancreatic tumours and prostate or brain cancers^14^, ^15^, ^16^, ^17^). In HCC, certain spliceosomal components are dysregulated and associated with liver oncogenesis, such as SF3B1, SRSF3, ESRP2 or MBNL3^18^; however, the expression profile of key spliceosome components and splicing factors has been only superficially explored in HCC.^19^, ^20^, ^21^ Therefore, this study aimed to comprehensively describe the pattern of dysregulation in the expression levels of a representative set of relevant spliceosome components and splicing factors and their relationship with clinical and molecular features, as well as their putative pathological role in HCC to further characterize the molecular basis underlying hepatic carcinogenesis.

## 2 MATERIALS AND METHODS

### 2.1 Patients, samples and cell lines

The study protocol was approved by the Reina Sofia University Hospital Ethics Committee, according to institutional and Good Clinical Practice guidelines (protocol number PI17/02287) and in compliment with the declaration of Helsinki. Informed consent was obtained from all patients or their relatives. Three independent cohorts of samples from patients with hepatic diseases were included: (1) Retrospective‐1: 172 formalin‐fixed paraffin‐embedded samples encompassing paired HCC and non‐tumour adjacent tissue (NTAT), (2) Retrospective‐2: snap‐frozen samples comprising HCC tissue (*n* = 57), NTAT (*n* = 47), cirrhotic liver samples (*n* = 41) and normal liver samples from autopsies (*n* = 5) and (3) Prospective‐1: plasma samples from HCC (*n* = 16), cirrhosis (*n* = 25), NAFLD (*n* = 28) patients and control individuals (*n* = 21). All these samples were obtained from the Andalusian Biobank (Cordoba Node). Liver tissues were evaluated by liver histology, and the diagnosis was confirmed by two independent, experienced pathologists. Clinical data from patients were collected from electronic medical reports. In silico analysis of HCC cohorts was performed as previously reported^18^ (Supporting Information). Liver cancer cell lines HepG2, Hep3B and SNU‐387 (HB‐8065) were used (ATCC, Manassas, USA). Cell line identity was validated by short tandem repeats sequences analysis. All cell lines were tested for mycoplasma by PCR, as previously reported^8^, ^18^, ^22^ (Supporting Information). The data that support the findings of this study are openly available in figshare at https://doi.org/10.6084/m9.figshare.16689061.v1.

### 2.2 Retrotranscription, PCR, qPCR, qPCR dynamic array, in vitro studies and western‐blot analysis

RNA isolation and retrotranscription, conventional PCR, qPCR and qPCR dynamic array have been previously reported.^13^, ^18^, ^22^ Measurements of cell proliferation, migration and formation of clones and tumourspheres have been performed as previously described.^18^ Western blotting has been previously reported.^18^, ^23^ More details about these approaches are provided in the Supporting Information section.

### 2.3 In vitro silencing/overexpression and pharmacological inhibition

Two small interfering RNAs for *EIF4A3* (*siEIF4A3#1*:ID138378; *siEIF4A3#2*:ID138379, Thermo Fisher) and a negative control (Scramble; Thermo Fisher) were used. For transfection, 120 000 SNU‐387 and 150 000 Hep3B or HepG2 cells were seeded in 6‐well plates.^18^ Medium was replaced by antibiotic/antimycotic‐free medium, and cells were transfected with 15 nM of each *siEIF4A3* siRNA (optimal concentration identified by dose‐response experiments of cell proliferation in HCC cell lines (Figure S3D)), using Lipofectamine RNAiMAX reagent (Thermo Fisher).

For EIF4A3 and FGFR4 overexpression, specific plasmids (pcDNA3.1+) were used. For transfection, 120 000 SNU‐387 and 150 000 Hep3B or HepG2 cells were seeded in 6‐well plates. Empty pCDNA3.1+ (mock transfected) was used as negative control. After 24 h, media were collected, and cells were detached and seeded to extract RNA and protein and to implement functional assays.

Moreover, cells were treated with a specific EIF4A3 inhibitor (EIF4A3‐IN‐1, 3 nM) (#HY‐101513, MedChemExpress, Monmouth Junction, NJ, USA) and a selective FGFR4 inhibitor (BLU9931 or BLU, 3 nM) (#HY‐12823, MedChemExpress).

### 2.4 EIF4A3 determination in plasma and cellular supernatant

Commercial ELISAs (MBS7234176, MyBioSource, San Diego, CA, USA) were used to determine plasma EIF4A3 levels in patients from Prospective‐1 cohort, and from supernatant of cell lines cultures, following the instructions of the manufacturer. The sensitivity of this assay is 1.0 pg/ml. No significant cross‐reactivity or interference between EIF4A3 and analogues has been reported. The donated plasma samples were stored in 1.5 ml aliquots at −80°C.

### 2.5 Xenograft model

Experiments were carried out according to the European Regulations for Animal Care under the approval of the University Research Ethics Committee. Eight‐week‐old nude male Fox1nu/Foxn1nu mice (Janvier Labs, Le Genest‐Saint‐Isle, France) were subcutaneously grafted in both flanks with 5 × 10^6^ Hep3B cells (*n* = 5 mice) in 50 μl of basement membrane extract (Trevigen, Gaithersburg, MD).^18^ Tumours growth and mice weight were monitored twice per week. Three weeks post grafting, when the tumours were visible, each tumour was locally treated with scramble or si*EIF4A3* using AteloGene (Koken, Tokyo, Japan), and tumour growth was monitored twice per week.^15^ After euthanasia, each tumour was dissected, and different pieces were snap frozen.

### 2.6 Statistical analysis

Data are expressed as mean ± standard error of the mean, as fold‐change (log 2) or relative levels compared with the corresponding controls (set at 100%). Data were evaluated for the heterogeneity of variance using the Kolmogorov–Smirnov test and, consequently, parametric (Student *t*) or nonparametric (Mann–Whitney *U*) tests were implemented. Spearman's or Pearson´s bivariate correlations were performed for quantitative variables according to normality. Significant relation between categorized mRNA expression and patient's survival was studied using Kaplan–Meier curves and long‐rank‐*p*. Statistical analysis of ROC curves, random forest and PLS‐DA analysis of mRNA expression from Retrospective‐1 cohort was performed using MetaboAnalyst 5.0. *p*‐Values lower than .05 were considered statistically significant. All statistics analyses were performed using the GraphPad Prism 6.0 software (La Jolla, CA, USA).

## 3 RESULTS

### 3.1 The expression landscape of spliceosome components and splicing factors is strongly dysregulated in HCC

**FIGURE 1:**
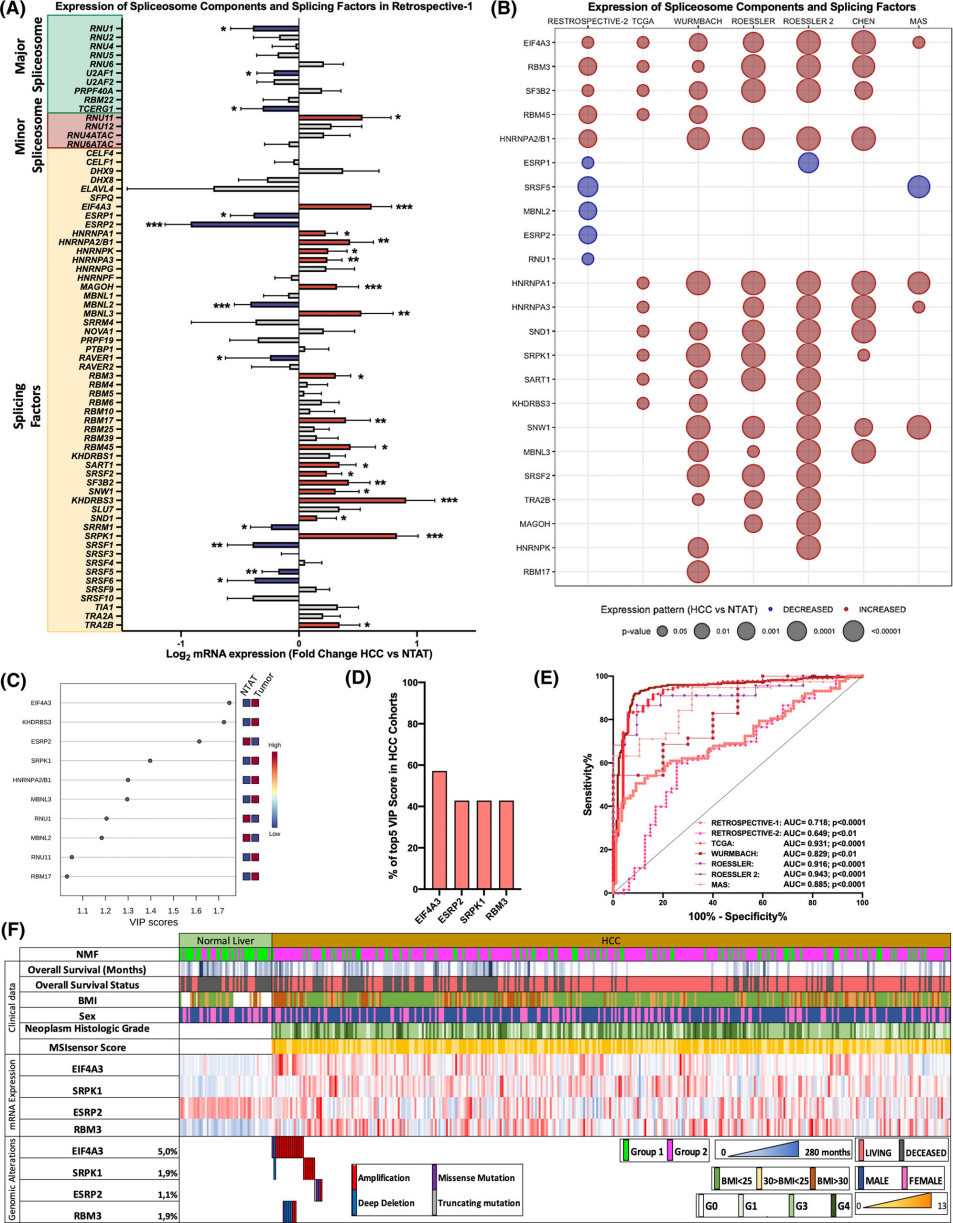
**Spliceosome components and splicing factors are altered in hepatocellular carcinoma (HCC)**: (A) fold‐change of spliceosome components and splicing factors expression in HCC tissue versus non‐tumour adjacent tissue (NTAT) in Retrospective‐1 cohort. Data are presented as mean ± standard error of the mean (SEM); (B) bubble plot of the expression pattern of significantly dysregulated spliceosome components and splicing factors in seven validation cohorts. The *y*‐axis indicates the spliceosome component and splicing factors altered in Retrospective‐1. Bubbles indicate the expression pattern. The bubble size indicates the *p*‐value; (C) VIP score analysis showing the spliceosome components and splicing factors with higher discriminatory capacity in Retrospective‐1; (D) spliceosomal components more frequently found among the five elements (Top 5) with more discriminatory capacity in all the studied cohorts by VIP score analysis; (E) ROC curve analysis constructed with the expression levels of *EIF4A3, ESRP2, SRPK1* and *RBM3* to discriminate between tumour and non‐tumour samples in all the studied cohorts; (F) transcriptomic and genomic alteration landscape of *EIF4A3, ESRP2, SRPK1* and *RBM3* in the TCGA cohort, and the clinical features of the patients. The asterisks (*p < .05; **p < .01; ***p < .001) indicate statistically significant differences

The systematic characterization of the spliceosomal landscape in HCC revealed that the expression of 30 out of 70 (42%) spliceosome components and splicing factors was significantly altered (11 decreased and 19 increased) in HCC compared to control NTAT from the discovery HCC cohort (Retrospective‐1) (Figure 1A; Table S1). These results were additionally analysed in another retrospective cohort (Retrospective‐2) (Table S1) and in six different in silico HCC cohorts compared to control tissues (Figure 1B). This analysis demonstrated that all the changes found in the discovery cohort were validated, at least, in a second cohort, and that 17 spliceosomal alterations were validated in more than 50% of the cohorts analysed (Figure 1B). Further analyses demonstrated that the expression pattern of spliceosomal components is clearly different between tumour and non‐tumour samples. This is indicated by PLS‐DA analysis (Figure S1A) that revealed that the discriminant capacity of the principal components is sufficient to assume a clear separation between the defined sample classes or by Random Forest classification that, using the significantly altered spliceosomal components, revealed an out‐of‐bag error of NTAT in .207 and tumour in .322 (Figure S1B). In addition, nonnegative matrix factorization consensus, an efficient method for distinct molecular patterns identification and powerful class discovery, classified TCGA patient in two groups based on their spliceosomal profile, which largely coincided with normal and tumour tissues (cophenetic coefficient = .9795; Figure S1C).

A VIP score analysis of Retrospective‐1 cohort revealed that the spliceosome components and splicing factors with more pronounced difference between HCC and NTAT (VIP score > 1.4) were *EIF4A3, KHDRBS3*, *ESRP2* and *SRPK1* (Figure 1C). When this VIP score analysis approach was implemented in all the retrospective and in silico cohorts, *EIF4A3*, *ESRP2*, *SRPK1* but also *RBM3* were the spliceosomal components more consistently present among the most discriminatory factors (Top 5 VIP scores) in all cohorts (Figures 1D and S1D). Multiple ROC curve analyses with these spliceosome components and splicing factors (*EIF4A3*, *RBM3*, *ESRP2* and *SRPK1*) were performed in all the cohorts, and the area under curve (AUC) obtained ranged from .649 to .943 (*p* < .01; Figure 1E). In addition, TCGA data also indicated that HCC is frequently associated with genomic alterations of these spliceosomal components (Figure S1C), including EIF4A3, RBM3, ESRP2 and SRPK1, which were altered in a 5%, 1.9%, 1.1% and 1.9% of the patients, respectively (Figure 1F). These genomic alterations were mostly amplifications in the case of EIF4A3, RBM3 and SRPK, which are overexpressed in HCC and deep deletions in the case of ESRP2, which is downregulated in HCC samples.

### 3.2 Expression of spliceosome components and splicing factors, especially EIF4A3, correlated with clinical features and oncogenic splicing variants

Expression of the spliceosomal elements more consistently altered in all the HCC cohorts (*EIF4A3*, *RBM3*, *ESRP2* or *SRPK1*) was associated with relevant clinical or molecular features of the patients. In particular, expression levels of *SRPK1, ESRP2* and *RBM3* were associated with lower survival rate (Figure S2A), with relevant clinical parameters such as microvascular invasion or with the expression of key oncogenic splicing variants (Figure S2B–D).

**FIGURE 2:**
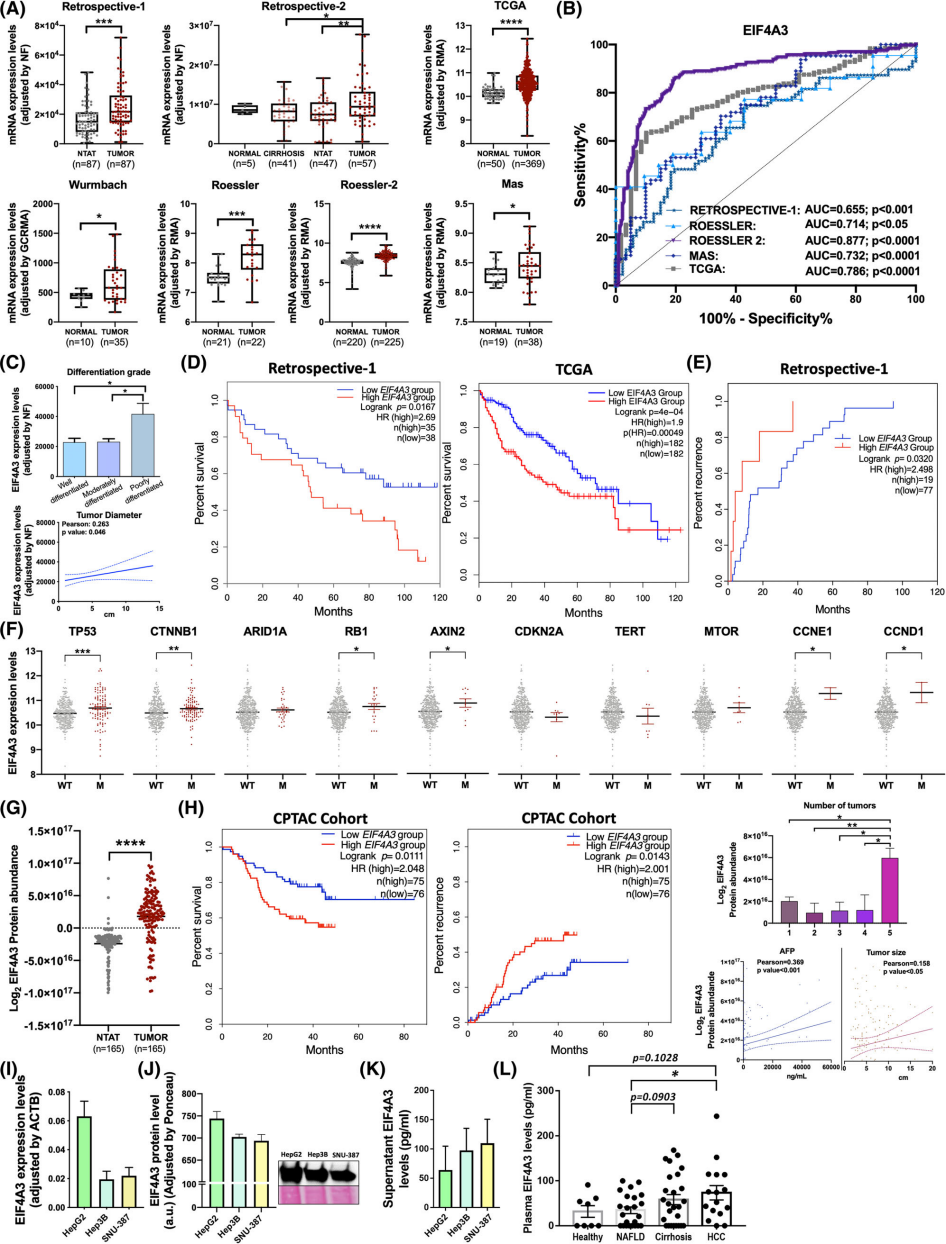
***EIF4A3* is associated with clinical aggressiveness and poor survival of hepatocellular carcinoma (HCC) patients**: (A) *EIF4A3* overexpression in HCC samples versus normal or non‐tumour adjacent tissue (NTAT) from seven different cohorts; (B) ROC curve analysis to discriminate between HCC versus normal or NTAT based on *EIF4A3* expression; (C) association between *EIF4A3* expression and clinical parameters in Retrospective‐1 cohort; (D) overall survival of patients from Retrospective‐1 and TCGA cohort categorized by the expression levels of *EIF4A3* (patients with highest expression vs. lowest expression group [cut‐off = median]) determined by long‐rank‐*p*‐value method; (E) recurrence of patients from Retrospective‐1 cohort (patients with highest expression vs. lowest expression group) determined by long‐rank‐*p*‐value method; (F) EIF4A3 expression levels in TCGA patients with mutations in key HCC genes; (G) EIF4A3 protein levels in CPTAC cohort; (H) overall survival and recurrence of patients from CPTAC cohort categorized by the protein levels of EIF3A3 (patients with highest expression vs. lowest expression group [cut‐off = median]) determined by long‐rank‐*p*‐value method, and association between EIF4A3 protein levels and clinical parameters in CPTAC cohort; (I) EIF4A3 mRNA levels in HepG2, Hep3B and SNU‐345 cell lines determined by qPCR and adjusted by ACTB expression; (J) EIF4A3 protein levels in HepG2, Hep3B and SNU‐345 cell lines determined by western‐blot; (K) EIF4A3 levels in supernatant from HepG2, Hep3B and SNU‐345 cells determined by ELISA; (L) EIF4A3 levels in plasma from a cohort of HCC (*n* = 16), cirrhosis (*n* = 25), NAFLD (*n* = 28) patients and control individuals (*n* = 21) determined by ELISA. The asterisks (*p < .05; **p < .01; ****p < .0001) indicate statistically significant differences. HR means hazard ratio

Among all the factors analysed, *EIF4A3* was significantly overexpressed in all the studied cohorts (Figure 2A). An ROC analysis revealed the significant discriminatory capacity of *EIF4A3* expression in five out of the seven cohorts with available data, with AUC ranging .655–.877 (Figure 2B). No differences in *EIF4A3* expression between aetiologies were observed in retrospective cohorts (Figure S2E,F). However, EIF4A3 levels were correlated with the expression of key oncogenic splicing variants in HCC samples from the retrospective cohorts (Figure S2G), and with important clinical features such as tumour differentiation and diameter (Figure 2C). Consistent with that, high levels of EIF4A3 were associated with lower survival in Retrospective‐1 and TCGA cohorts (Figure 2D), as well as with higher recurrence in HCC patients of Retrospective‐1 cohort (Figure 2E). Interestingly, EIF4A3 levels were significantly higher in TCGA patients with mutations in key HCC genes, such as TP53, CTNNB1, RB1, AXIN2, CCNE1 or CCND1 (Figure 2F). Additionally, higher EIF4A3 protein levels were observed in HCC samples from the CPTAC cohort compared to NTAT (Figure 2G), associated with lower survival and higher recurrence, and correlated with the number of tumours, alpha‐fetoprotein levels and tumour size (Figure 2H).

Consistently, the three liver cancer cell lines studied here exhibited considerable EIF4A3 expression at mRNA (Figure 2I) and protein (Figure 2J) levels and were able to release it (Figure 2K). To investigate the diagnostic capacity of EIF4A3 in plasma samples, we evaluated EIF4A3 levels in patients with NAFLD, cirrhosis and HCC, as well as control individuals (Prospective‐1 cohort; Table S2). Plasma EIF4A3 levels were higher in HCC patients compared to controls and NAFLD patients (Figure 2L), suggesting that EIF4A3 might serve as an early biomarker for HCC development.

### 3.3 *EIF4A3*‐silencing reduced aggressiveness features of liver cancer cell lines in vitro and in vivo

**FIGURE 3:**
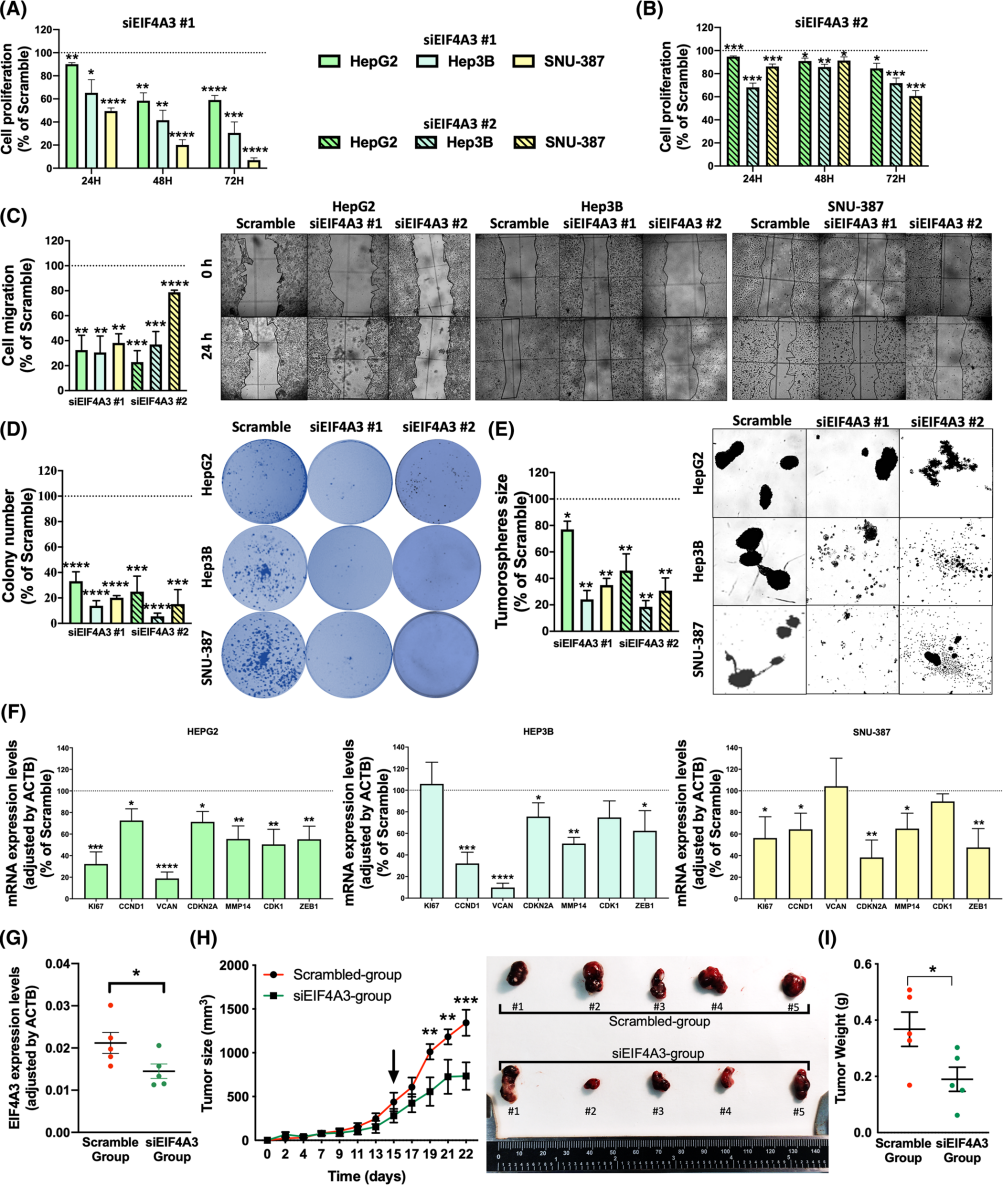
***EIF4A3* silencing decreases aggressiveness of hepatocellular carcinoma (HCC) cells**: proliferation of *EIF4A3*‐silenced with *siEIF4A3#1* (A) and *siEIF4A3#2* (B) compared to scramble‐treated cell lines (HepG2, Hep3B and SNU‐387) at 24, 48 and 72 h determined by the Alamar Blue assay; (C) migration of *EIF4A3*‐silenced compared to scramble‐treated cells. Representative images of cell migration after 24 h are depicted; (D) number of colonies formed in *EIF4A3*‐silenced compared to scramble‐treated cells. Representative images of colonies formed after 10 days are depicted; (E) mean tumoursphere size of *EIF4A3*‐silenced compared to scramble‐treated cells. Representative images of tumourspheres formed after 10 days are depicted; (F) mRNA expression levels of key tumour markers genes in EIF4A3‐silenced versus scramble‐treated cells; (G) Validation of *EIF4A3* expression by qPCR after in vivo silencing in xenograft models; (H) growth rate of tumours in Hep3B‐induced xenograft tumours in nude mice (*n* = 5) before and after in vivo *EIF4A3*‐silencing (indicated by the arrow). Representative images of scramble‐ and siEIF4A3‐treated tumours are depicted; (I) final tumour weight of scramble‐ and siEIF4A3‐treated tumours. Data are presented as mean ± standard error of the mean (SEM) from *n* = 3–5 independent experiments. The asterisks (*p < .05; **p < .01; ***p < .001; ****p < .0001) indicate statistically significant differences

Reduction in the expression levels of *EIF4A3* using two specific siRNAs, *siEIF4A3#1 and siEIF4A3#2* (confirmed at mRNA and protein levels; Figure S3A,B), induced a dose‐dependently inhibition of cell proliferation at 24, 48 and 72 h (*siEIF4A3#1*:HepG2 [at 72 h, 41%, *p*‐value = .0005], Hep3B [at 72 h, 69.47%, *p*‐value = .002], SNU‐387 [at 72 h, 93.04%, *p*‐value < .0001]; *siEIF4A3#2*:HepG2 [at 72 h, 15.41%, *p*‐value = .03], Hep3B [at 72 h, 28.17%, *p*‐value = .0007], SNU‐387 [at 72 h, 39.32%, *p*‐value = .0002]) (Figures 3A,B and S3C). In addition, wound‐healing assays demonstrated that both *siEIF4A3s* significantly reduced the migration capacity (*siEIF4A3#1*:HepG2 [67.55%, *p*‐value = .0012], Hep3B [69.45%, *p*‐value = .0063], SNU‐387 [61.80%, *p*‐value = .0011]; *siEIF4A3#2*:HepG2 [77.09%, *p*‐value = .0001], Hep3B [63.01%, *p*‐value = .0009], SNU‐387 [21.23%, *p*‐value = .0001]) (Figure 3C), whereas clonogenic assays showed that the number of colonies formed was significantly lower in response to both *siEIF4A3s* (*siEIF4A3#1*:HepG2 [66.85%, *p*‐value < .0001], Hep3B [86.11%, *p*‐value < .0001], SNU‐387 [79.81%, *p*‐value < .0001]; *siEIF4A3#2*:HepG2 [75.09%, *p*‐value < .0001], Hep3B [94.43%, *p*‐value < .0001], SNU‐387 [84.88%, *p*‐value < .0001]) (Figure 3D). Similarly, the mean size of tumourspheres was also markedly reduced in response to *siEIF4A3s* (*siEIF4A3#1*:HepG2 [22.92%, *p*‐value = .02], Hep3B [75.81%, *p*‐value < .0077], SNU‐387 [65.06%, *p*‐value = .0061]; *siEIF4A3#2*:HepG2 [54.04%, *p*‐value = .0009], Hep3B [81.47%, *p*‐value < .0001], SNU‐387 [69.27%, *p*‐value = .0003]) (Figure 3E). Moreover, invasion assay showed a reduction of invasion capacity of EIF4A3‐silenced Hep3B and SNU‐387 cells in comparison with scramble‐treated cells (*siEIF4A3#1*:Hep3B [89.68%, *p*‐value < .0001], SNU‐387 [93.91%, *p*‐value < .0001]) (Figure S3D). These functional alterations were associated with changes in the expression of different tumour markers (CCND1, CDKN2A, etc.; determined by qPCR), which were modulated in a cell‐dependent manner in response of EIF4A3‐silencing (Figure 3F).

Consistent with these results, in vivo silencing of *EIF4A3* (validated at mRNA levels by qPCR; Figure 3G) in Hep3B‐induced xenografts also reduced in vivo xenograft tumours growth in nude mice. In particular, *EIF4A3*‐silencing in vivo in established subcutaneous tumours significantly reduced tumour growth (*p*‐value < .0001) (Figure 3H) and final tumour weight (Figure 3I) compared to scramble‐treated tumours (*p*‐value < .04).

### 3.4 Pharmacologic blockade and overexpression of *EIF4A3* altered the behaviour of liver cancer cells in vitro

**FIGURE 4:**
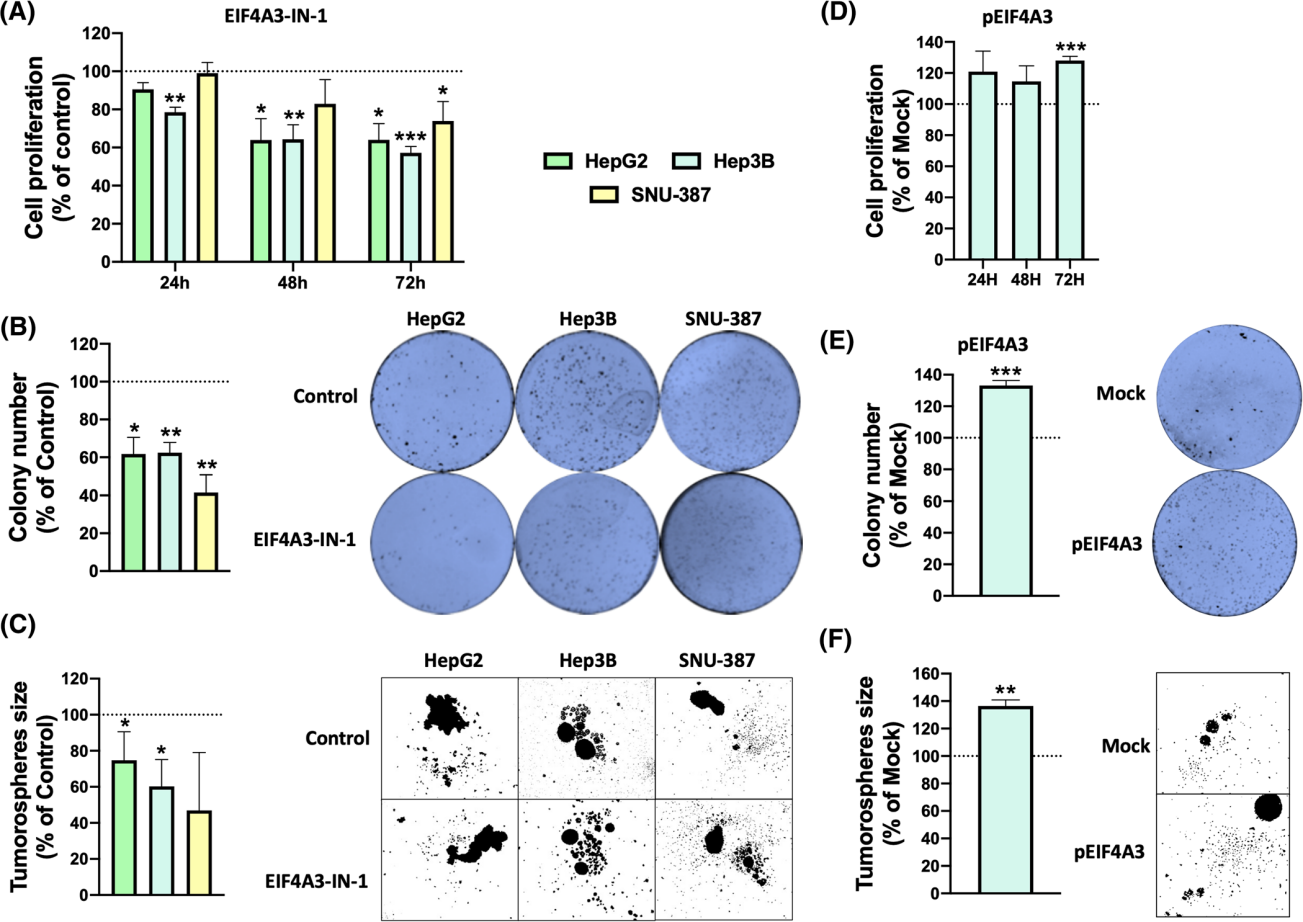
**Pharmacologic inhibition or overexpression of EIF4A3 alter aggressiveness of liver cancer cells**: (A) cell proliferation was determined in EIF4A3‐IN‐1 and vehicle‐treated HepG2, Hep3B and SNU‐387 cells by the Alamar Blue assay at 24, 48 and 72 h; (B) number of colonies formed in EIF4A3‐IN‐1 treated cells compared to vehicle‐treated cells. Representative images of colonies formed after 10 days are depicted; (C) mean tumoursphere size of in EIF4A3‐IN‐1 treated cells compared to vehicle‐treated cells. Representative images of tumourspheres formed after 10 days are depicted; (D) cell proliferation was determined in pEIF4A3‐transfected HepG2 cells in comparison with mock cells by the Alamar Blue assay at 24, 48 and 72 h; (E) number of colonies formed in pEIF4A3‐transfected cells compared to mock cells. Representative images of colonies formed after 10 days are depicted; (F) mean tumoursphere size of in pEIF4A3‐transfected cells compared to mock cells. Representative images of tumourspheres formed after 10 days are depicted. Data are presented as mean ± standard error of the mean (SEM) from *n* = 3–5 independent experiments. Asterisks (*p < .05; ***p < .001) indicate statistically significant differences versus scramble‐treated controls. Dashes (#*p* < .05; ##*p* < .01; ###*p* < .001) indicate statistically significant differences versus vehicle‐treated controls

Consistent with the reduction in the aggressiveness of liver cancer cells in response to *EIF4A3*‐silencing, the pharmacologic blockade of EIF4A3 (using EIF4A3‐IN‐1, a selective inhibitor of EIF4A3) significantly reduced cell proliferation, colony formation and tumourspheres size in the three liver cancer cell lines (cell proliferation: HepG2 [at 72 h, 35.92%, *p*‐value = .013], Hep3B [at 72 h, 42.75%, *p*‐value = .0002], SNU‐387 [at 72 h, 26.10%, *p*‐value = .028]; colony formation: HepG2 [38.35%, *p*‐value = .0121], Hep3B [37.58%, *p*‐value = .0022], SNU‐387 [58.44%, *p*‐value = .0033]; tumourspheres size: HepG2 [25.28%, *p*‐value = .0163], Hep3B [39.73%, *p*‐value = .0244], SNU‐387 [53.06%, *p*‐value = .077]) (Figure 4A–C) in comparison with vehicle‐treated cells.

As a proof‐of‐concept, the forced overexpression of EIF4A3 in Hep3B cells by EIF4A3‐pcDNA3.1 (pEIF4A3; confirmed at mRNA and protein levels; Figure S3E,F) increased cell proliferation, colony formation and tumourspheres size (cell proliferation: Hep3B [at 72 h, 28.07%, *p*‐value = .0004]; colony formation: Hep3B [31.92%, *p*‐value = .0004], tumourspheres size: Hep3B [36.44%, *p*‐value = .0012]) (Figure 4D–F) in comparison with empty pcDNA3.1‐transfected cells (mock).

### 3.5 *EIF4A3* impacts transcription and splicing landscape of critical genes in HCC

**FIGURE 5:**
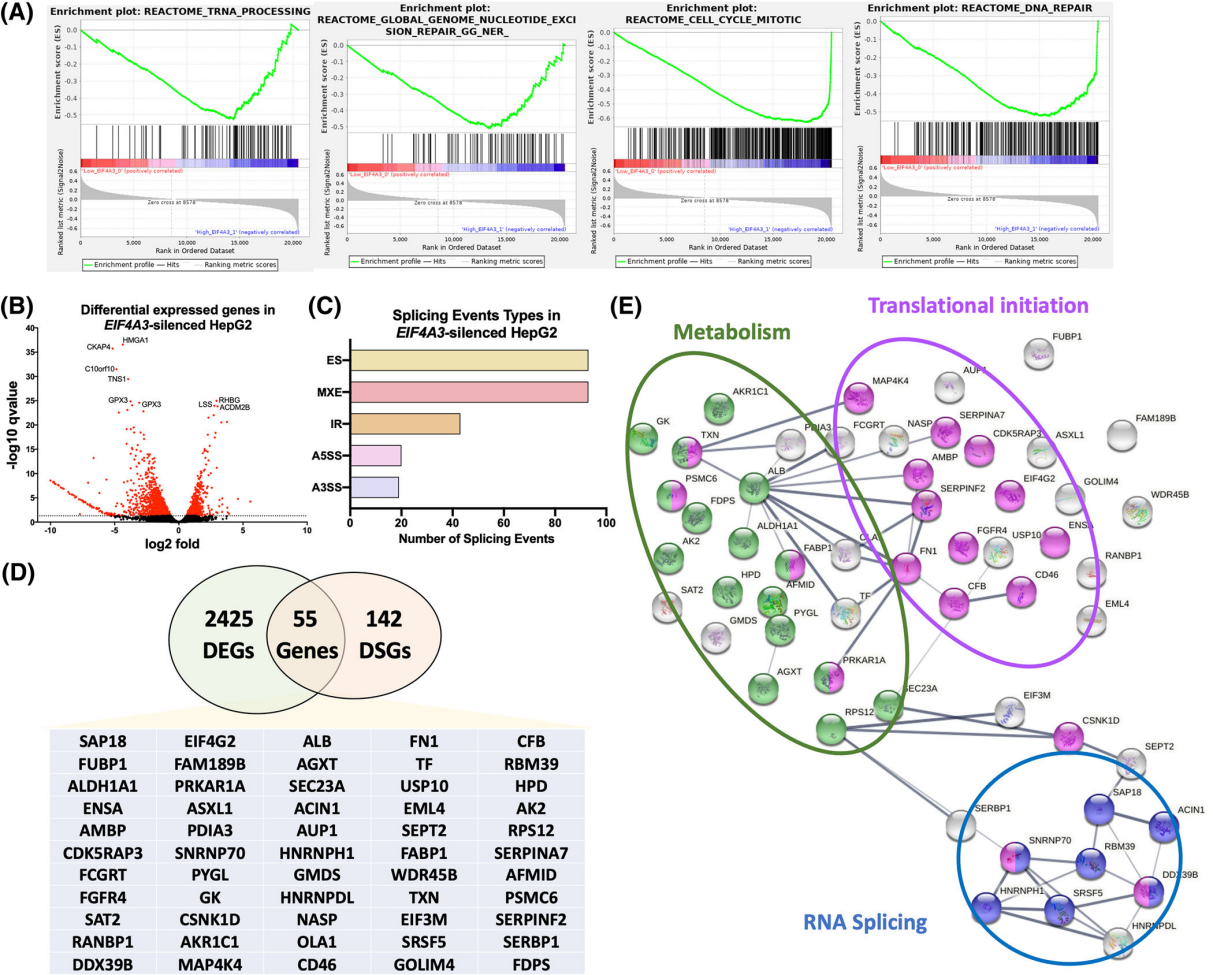
**EIF4A3 associates with the expression and splicing of key hepatocellular carcinoma (HCC)‐related genes**: (A) GSEA analysis performed by GenePattern in Reactome using the TCGA cohort classified by *EIF4A3* expression levels in low and high EIF4A3 groups; (B) differentially expressed genes (DEGs) in *EIF4A3*‐silenced HepG2 cells obtained from RNAseq data (FDR < .05); (C) splicing event types in *EIF4A3*‐silenced HepG2 cells obtained from RNAseq data (FDR < .05); (D) the Venn diagram of DEGs and differentially spliced genes (DSGs) in *EIF4A3*‐silenced HepG2 cells (FDR < .05); (E) STRING analysis of the 55 genes with differential expression and splicing pattern in *EIF4A3*‐silenced HepG2 cells (FDR < .05)

GSEA analysis performed by GenePattern in Reactome using the TCGA cohort and classifying by *EIF4A3* expression levels in low and high *EIF4A3* groups demonstrated a tight association of *EIF4A3* with specific molecular signatures. Interestingly, the high *EIF4A3* group was enriched in tumour‐related pathways such as tRNA processing, nucleotide excision repair, cell cycle and DNA repair (Figure 5A). To gain further insight, we analysed available RNAseq data from *EIF4A3*‐silenced HepG2 cells, identifying the dysregulation of the expression of 2480 genes (FDR < .05) (Figure 5B). In addition, the silencing of *EIF4A3* induced the alteration of 293 splicing events in 197 genes (FDR < .05), wherein most splicing events were exon skipping (ES) and mutually exclusive exons (Figure 5C). A significant number of these splicing events were validated in vitro using *EIF4A3*‐silenced HepG2 cells, which showed that the splicing pattern of some of these relevant genes (*ACIN1*, *ASLX1*, *CD5KRAP3* or *PYGL*) was altered after *EIF4A3*‐silencing (Figure S4A,B), confirming the implication of EIF4A3 in the splicing of crucial genes in HCC pathophysiology. The intersection of the 2480 differentially expressed genes with the 197 differentially spliced genes revealed the existence of 55 genes with differential expression and splicing pattern in HepG2 cells treated with *siEIF4A3* (Figure 5D). Notably, the STRING analysis of these 55 genes with differential expression and altered splicing pattern revealed the existence of 3 gene clusters implicated in RNA splicing, metabolism and translational initiation (Figure 5E), the former one including relevant genes in HCC such as *FGFR4*, the FGF19 receptor, which has been reported to exert oncogenic roles in HCC.^24^

### 3.6 *FGFR4* expression, splicing and function is directly modulated by EIF4A3

**FIGURE 6:**
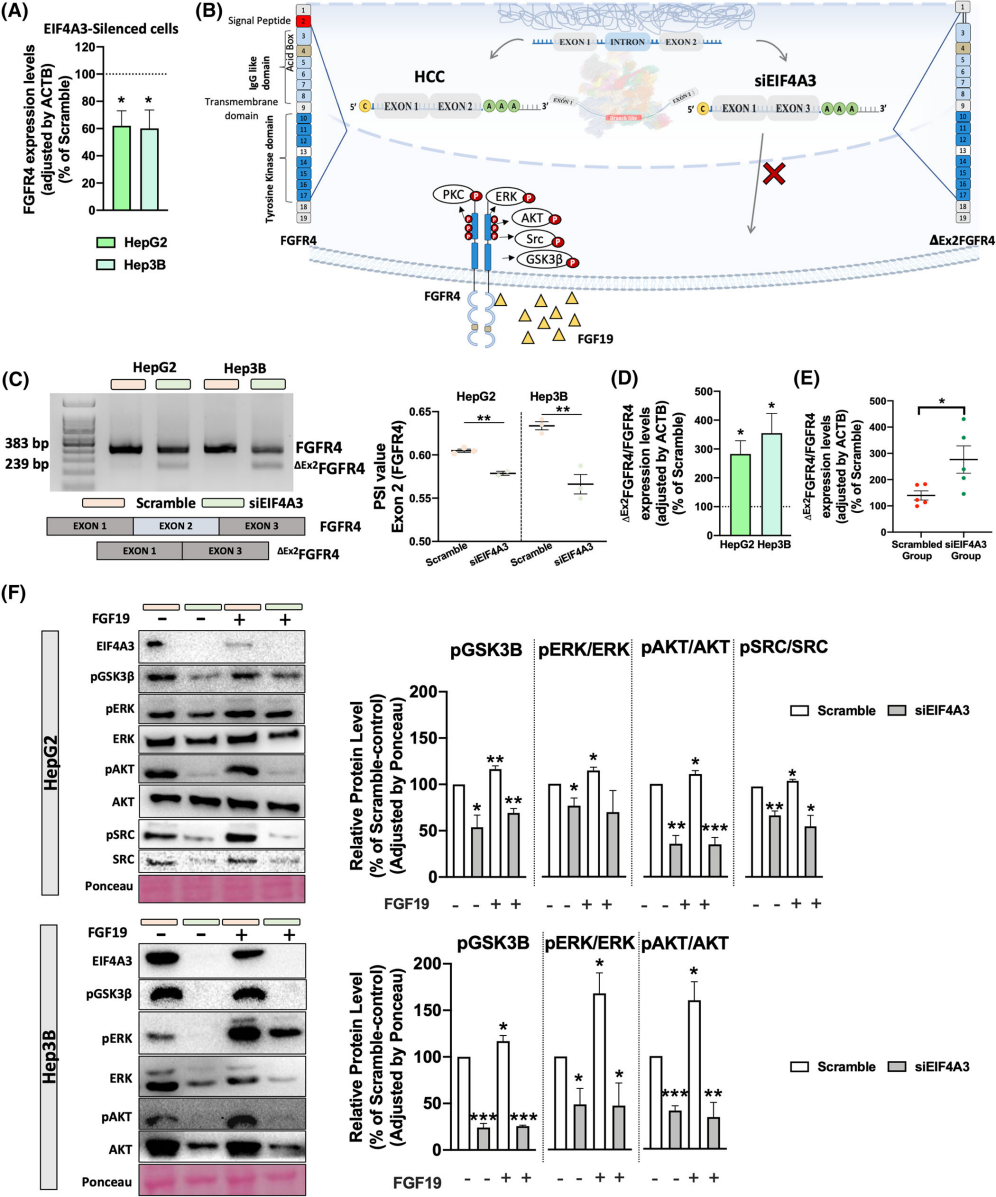
**EIF4A3 modulates FGFR4 expression and splicing and reduces functional signalling of FGF19/FGFR4**: (A) FGFR4 expression levels determined by qPCR in *EIF4A3*‐silenced cells (HepG2 and Hep3B) compared with scramble‐treated controls; (B) working hypothesis showing the implication of *EIF4A3* silencing on *FGFR4* exon 2 skipping; (C) validation of FGFR4 exon 2 skipping event in HepG2 and Hep3B cells in response to *EIF4A3* silencing by qPCR. PSI means per cent spliced in; (D) validation of *FGFR4* exon 2 skipping event calculated by the expression rate of ^dEx2^FGFR4 and full‐length FGFR4 in response to *EIF4A3*‐silencing in HepG2 and Hep3b cells by qPCR; (E) validation of *FGFR4* exon 2 event skipping calculated by the expression rate of *^dEx2^FGFR4* and full‐length *FGFR4* in response to *EIF4A3* silencing in Hep3B‐induced and scramble‐ or *siEIF4A3*‐treated xenograft tumours by qPCR; (F) Western‐blot of downstream signalling of FGFR4 in HepG2 and Hep3B cells in response to *EIF4A3*‐silencing alone or in combination with FGF19 exogenous treatment (100 nM). Relative protein level for pGSK3B, pERK, pAKT and pSRC, normalized to total protein, respectively, and all protein level were normalized by Ponceau. Data are presented as mean ± standard error of the mean (SEM) from *n* = 3–5 independent experiments. Asterisks (*p < .05; **p < .01; ***p < .001; ****p < .0001) indicate statistically significant differences

In that RNAseq data from *EIF4A3*‐silenced HepG2 cells unveiled that *EIF4A3*‐silencing significantly altered the expression levels and splicing pattern of *FGFR4*,^24^ we next aimed to validate this modulation of *FGFR4* expression by EIF4A3 in vitro in HepG2 and Hep3B cells, wherein the silencing of *EIF4A3* reduced *FGFR4* expression (Figure 6A). In the case of the splicing pattern, RNAseq data from EIF4A3‐silenced HepG2 cells revealed two splicing events in FGFR4 gene in response to EIF4A3‐silencing: ES of exon 2 (IncLevelDifference: −.533; *p*‐value: .029) and intron retention of intron 3 (IncLevelDifference: .024; *p*‐value: .00004) (Figure S4C). Because the exon 2 encodes the signal peptide of FGFR4, we further focused on this ES event by hypothesizing that low *EIF4A3* expression levels could reduce the inclusion of the exon 2, and therefore, depriving the FGFR4 from its signal peptide, thus, reducing the oncogenic potential of the FGF19/FGFR4 pathway (Figure 6B).

The role of EIF4A3 on the ES of *FGFR4* exon 2 was validated in vitro and in vivo. First, conventional PCR in HepG2 and Hep3B cells demonstrated that *EIF4A3*‐silencing reduced the inclusion of exon 2 and induced the appearance of the exon 2 skipped variant *^dEx2^FGFR4* (Figure 6C). Indeed, the PSI value of the exon 2 skipping event was significantly reduced in both cell lines (Figure 6C). Consistently, qPCR confirmed higher rate of *^dEx2^FGFR4*/*FGFR4* in response to *EIF4A3*‐silencing in both cell lines treated in vitro (Figure 6D), as well as in the Hep3B‐induced in vivo tumours formed in nude mice (Figure 6E).

Furthermore, we demonstrated that the EIF4A3‐dependent inclusion of exon 2 in *FGFR4* is essential to mediate the signalling of FGF19 and the maintaining of the FGF19/FGFR4 axis in HCC, in that *EIF4A3*‐silencing not only reduced the basal phosphorylation levels of downstream pathways associated to FGF19/FGFR4, including GSK3β(Ser9), ERK(Thr202/Tyr204), AKT(Ser473) and SRC(Y419) (Figure 6F), but also blunted the FGF19‐induced phosphorylation of GSK3β, ERK, AKT and SRC in liver cancer cells (Figure 6F). Remarkably, the activation of these signalling pathways was not fully compromised in EIF4A3‐silenced cells in that other ligand such as insulin (100 nM) induced a similar or even higher level of phosphorylation of GSK3β, ERK and AKT in scramble‐ and *EIF4A3*‐silenced cells (Figure S5A,B), suggesting a selective role of EIF4A3 in the modulation of FGF19/FGFR4 pathway.

### 3.7 The functional consequences of *EIF4A3*‐silencing are mediated through the modulation of *FGFR4* splicing

**FIGURE 7:**
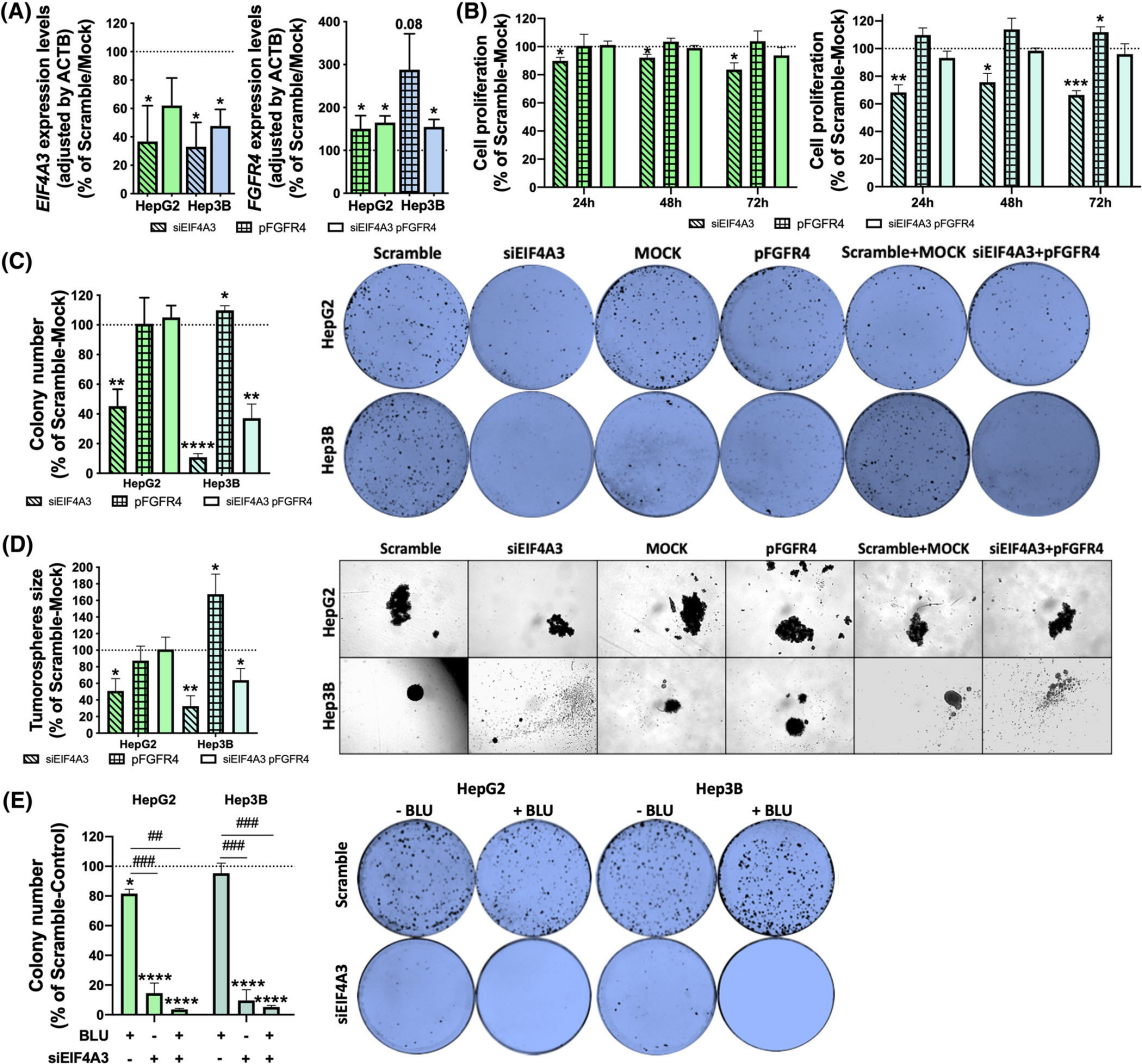
**EIF4A3 silencing exerts its inhibitory actions by modulating FGFR4 splicing**: (A) validation of *EIF4A3* and *FGFR4* expression in rescue experiments. EIF4A3 was silenced alone or in combination with FGFR4 overexpression in HepG2 and Hep3B cells, and the expression of both genes was validated in all the experimental conditions by qPCR; (B) cell proliferation determined in siEIF4A3‐treated, pFGFR4‐transfected and siEIF4A3‐treated/pFGFR4‐transfected, compared with scramble‐treated/mock HepG2 and Hep3B cells by Alamar Blue assay at 24, 48 and 72 h; (C) number of colonies formed in siEIF4A3‐treated, pFGFR4‐transfected and siEIF4A3‐treated/pFGFR4‐transfected, compared with scramble‐treated/mock HepG2 and Hep3B cells. Representative images of colonies formed after 10 days are depicted; (D) mean tumoursphere size of siEIF4A3‐treated, pFGFR4‐transfected and siEIF4A3‐treated/pFGFR4‐transfected, compared with scramble‐treated/mock HepG2 and Hep3B cells. Representative images of tumourspheres formed after 10 days are depicted; (E) number of colonies formed in si*EIF4A3*‐treated, BLU‐treated and si*EIF4A3*‐treated/BLU‐treated HepG2 and Hep3B cells compared to scramble‐ treated/control cells. Representative images of colonies formed after 10 days are depicted. Data are presented as mean ± standard error of the mean (SEM) from *n* = 3–5 independent experiments. Asterisks (*p < .05; **p < .01; ***p < .001; ****p < .0001) indicate statistically significant differences versus scramble‐treated or mock controls, whereas dashes (^##^ *p* < .01; ^###^ *p* < .001) indicate statistically significant differences versus vehicle‐treated controls

To unveil the implication of *FGFR4* splicing in the functional role of EIF4A3 in HCC, we performed a rescue assay overexpressing the full‐length FGFR4 receptor (which is a FGFR4 version not susceptible to be processed through splicing as long as it does not contain introns) in EIF4A3‐silenced cells (HepG2 and Hep3B; Figure 7A). This approach revealed that, in contrast to *EIF4A3*‐silencing that clearly reduces the proliferation rate of HepG2 and Hep3B cells (HepG2 [at 72 h, 16.37%, *p*‐value = .0282], Hep3B [at 72 h, 33.59%, *p*‐value = .0004]), the overexpression of FGFR4 does not alter or slightly increased the proliferation rate of these liver cells (HepG2 [at 72 h, 3.89%, *p*‐value = .6185], Hep3B [at 72 h, 11.96%, *p*‐value = .035]) (Figure 7B). Remarkably, the overexpression of full‐length FGFR4 receptor in EIF4A3‐silenced cells rescued the parental phenotype of the cells as they were resistant to the inhibition of the proliferation rate induced by EIF4A3‐silencing (HepG2 [at 72 h, 6.28%, *p*‐value = .3275], Hep3B [at 72 h, 1.74%, *p*‐value = .8211]) (Figure 7B). Similarly, FGFR4 overexpression, which did not alter colony and tumoursphere formation in HepG2 cells, completely rescued the reduction of these parameters induced by *EIF4A3*‐silencing (colony formation: [siEIF4A3 + pFGFR4, 5.05%, *p*‐value = .5614], tumoursphere formation [siEIF4A3 + pFGFR4, .87%, *p*‐value = .9564]) (Figure 7C,D). In the case of Hep3B, FGFR4 slightly increased the capacity to form colonies and tumourspheres and partially rescued the cells from the inhibition induced by *EIF4A3*‐silencing (colony formation: [siEIF4A3 + pFGFR4, 62.83%, *p*‐value = .0027]; tumoursphere formation: [siEIF4A3 + pFGFR4, 36.14%, *p*‐value = .06]) (Figure 7C,D). Overall, these results demonstrate that the functional consequences observed after EIF4A3‐silencing are, at least in part, mediated by the dysregulation of FGFR4 splicing.

To further validate this idea and to explore the putative utility of blocking EIF4A3 and FGFR4 simultaneously in liver cancer cells, we performed functional assays in response to EIF4A3‐silencing alone or in combination with a specific FGFR4‐inhibitor (BLU9931 or BLU). These studies showed that the number of colonies formed was slightly lower in response to BLU or were profoundly reduced in response to *siEIF4A3#1* in comparison with scramble‐control cells (BLU: HepG2 [13.09%, *p*‐value = .0394], Hep3B [12.04%, *p*‐value = .3380]; *siEIF4A3#1*:HepG2 [85.81%, *p*‐value < .0001], Hep3B [91.09%, *p*‐value < .0001]) (Figure 7E). Remarkably, the combination of *siEIF4A3#1* and the FGFR4 inhibitor did not exerted more pronounced effects (Figure 7E), which further indicate that EIF4A3 may act in HCC cells through the control of FGF19/FGFR4 signalling.

## 4 DISCUSSION

The present study provides original and compelling data demonstrating that a high proportion of spliceosomal elements is altered in HCC in comparison with NTAT, including key spliceosome components and splicing factors, such as EIF4A3, ESRP2, SRPK1 and RBM3, which were consistently validated in seven additional in silico cohorts (at mRNA or protein levels). An important proportion of these spliceosomal elements was altered in most of the HCC cohorts, comprising a spliceosome‐related molecular fingerprint with diagnostic, prognostic, and therapeutic implications. Indeed, the expression of these elements, especially *EIF4A3*, was associated with key clinical and aggressiveness parameters and, most importantly, with patient overall recurrence and survival, suggesting a putative implication of the dysregulations of the splicing machinery and the development and progression of HCC. Even more, our results demonstrate that different liver cell lines express and release EIF4A3, and that plasma EIF4A3 levels were significantly higher in HCC patients compared to non‐HCC controls (healthy and NAFLD patients), demonstrating the diagnostic capacity of EIF4A3 levels in liquid biopsy. This study also demonstrates that EIF4A3 can control tumourigenic capacity of liver cancer cells in vitro and the in vivo tumour growth in a preclinical HCC model of Hep3B‐induced xenografts by the alteration of the expression and splicing events of key oncogenes such as *FGFR4*.

These results, therefore, reinforce our previous knowledge on the dysregulation of the spliceosomal landscape in cancer cells^10^, ^25^, ^26^ and provide a solid evidence of novel and relevant splicing‐related elements (i.e. EIF4A3) that are consistently altered and exert an important role in HCC, which further strengthen the relationship and implication of the splicing process and the development and progression of HCC. Indeed, ESRP2, SRPK1 and RBM3 have a crucial role in maintaining the appropriate splicing process, and their dysregulation has been described and associated with worse clinical characteristics in tumour pathologies, including HCC.^27^, ^28^, ^29^ Consistent with this idea, recent studies suggest that certain spliceosome components and/or splicing factors could represent novel biomarkers and/or therapeutic targets in HCC, and that their expression levels can have a potential utility as prognostic markers.^18^ This is further supported by the fact that cancer cells seem to be highly sensitive to a reduction in spliceosomal activity, whereas normal cells seem to tolerate a reduction in the activity of certain spliceosome components,^18^ paving the way towards the targeting of the splicing machinery to develop novel strategies in the management and treatment of HCC. In this sense, we have previously demonstrated that the modulation of the activity of the spliceosome through the inhibition of a specific component has a potential therapeutic application in certain tumour pathologies,^30^, ^31^, ^32^ including HCC.^18^ However, most drugs directed to modulate the splicing process have been designed to target SF3B1, which suggests the necessity of exploring additional targets among spliceosome components and splicing factors that exhibit consistent dysregulation in tumour pathologies and that could represent novel approaches to improve the management of this pathology.

Importantly, this study is the first to characterize the role of EIF4A3 in HCC. EIF4A3 is, together with RBM8A and MAGOH, the main RNA‐binding components of the exon junction complex, a multi‐protein complex involved in mRNA metabolism.^33^ This factor is an important regulator of post‐transcriptional processes, including mRNA splicing, transport, translation and surveillance. The role of EIF4A3 has been described in some tumour pathologies such as glioblastoma multiforme or breast cancer, wherein EIF4A3 could facilitate circMMP9 and circSEPT9 cyclization, facilitating carcinogenesis.^34^, ^35^ Consistently, EIF4A3 has been shown to be altered in HCC samples in in silico–based studies.^36^, ^37^ However, the association with clinical characteristics, the clinical implications, the molecular mechanisms and the role in HCC were still to be elucidated. In this sense, our data demonstrate that EIF4A3 is overexpressed at mRNA and protein level in all the HCC cohorts explored, which indicates that EIF4A3, in contrast with other spliceosomal components and splicing factors analysed, may represent a universal hallmark in HCC development and progression. In addition, we have also found that higher EIF4A3 expression is associated with the presence of classic HCC mutations, such as TP53 or CTNNB1. These mutations are crucial in HCC development and are associated with clinical parameters of the patients and with particular expression patterns in tumour samples. Although the implication of these mutations in the expression of EIF4A3 is not clear and should be further explored in future studies, these results provide valuable and clinically relevant information to understand the impact of dysregulated EIF4A3 in HCC samples and its implication in the development and progression of this cancer type. Consistent with that, our results also demonstrate that higher EIF4A3 expression is associated with higher recurrence and worse survival in patients with HCC.

Moreover, the in vitro modulation of *EIF4A3* expression with specific siRNAs or its pharmacological inhibition decreased key functional parameters of aggressiveness, including proliferation, migration, invasion, tumoursphere size and colony formation in all liver cancer cell lines analysed and reduced tumour growth in a preclinical model (Hep3B‐induced xenograft tumours). As a proof‐of‐concept, the forced overexpression of EIF4A3 increased the tumourigenic properties of liver cancer cells, demonstrating the implication of EIF4A3 in HCC biology. This is consistent with that found in other pathologies,^34^, ^35^ suggesting a crucial role of EIF4A3 dysregulations in cancer development and/or progression.

Mechanistically, EIF4A3 is involved in the modulation of multiple key targets and molecular pathways. Indeed, in the TCGA cohort, HCC samples with high *EIF4A3* levels were enriched in key tumour‐related pathways, such as tRNA processing, nucleotide excision repair, cell cycle mitotic and DNA repair.^38^, ^39^ More detailed analysis of RNAseq data from *EIF4A3*‐silenced HepG2 cells confirmed that the expression levels and the splicing pattern of numerous genes are profoundly altered in response to *EIF4A3*‐silencing. In particular, the analysis of the genes with differential expression and altered splicing pattern revealed the existence of three gene clusters related with RNA splicing, metabolism and translational initiation, the former one including relevant genes in HCC such as *FGFR4*. Indeed, our results demonstrate that EIF4A3 can control the expression and splicing of *FGFR4*. The role of FGFR4 and its ligand, FGF19, in regulating cellular proliferation, differentiation and angiogenesis are well known.^24^ Specifically in HCC, FGFR4 and FGF19 are overexpressed and play a key role in hepatocarcinogenesis, metastasis and drug resistance.^40^, ^41^, ^42^ Importantly, our study demonstrates that the silencing of *EIF4A3* significantly altered the splicing pattern of *FGFR4*, leading to the skipping of exon 2, which encodes the signal peptide responsible for the translocation of FGFR4 to the cell membrane. These results therefore indicate that EIF4A3 is necessary for the appropriate splicing process of *FGFR4*. Indeed, *EIF4A3*‐silencing abrogated FGF19 signalling in liver cancer cells in terms of the phosphorylation of key FGFR4‐downstream effectors (AKT, ERK, SRC and GSK3B),^24^ thus suggesting a role of this splicing factor in sustaining the FGF19/FGFR4 oncogenic pathway. In addition, rescue experiments demonstrated that the forced expression of a non‐spliceable version of the full‐length FGFR4 in *EIF4A3*‐silenced cells completely (or partially) restored the inhibitory effects of *EIF4A3*‐silenced. Consistently, our data also indicate that the blockade of the FGF19/FGFR4 pathway by small molecules (i.e. irreversible FGFR4 inhibitors), which is currently being evaluated in clinical trials,^43^ cannot further exacerbate the inhibitory effect induced by *EIF4A3*‐silencing, thus showing that the main actions exerted by EIF4A3 in the modulation of HCC aggressiveness are mediated by the controlling of FGFR4 splicing.

In conclusion, our results provide novel and compelling evidence to support that the cellular machinery that regulates the splicing process (spliceosome components and splicing factors) is strongly dysregulated in HCC, and that certain spliceosome components (EIF4A3, ESRP2, SRPK1 and RBM3) could provide novel diagnostic and prognostic biomarkers and therapeutic targets in HCC. Indeed, EIF4A3 emerged as an actionable splicing factor as its expression is consistently elevated in HCC and associated with increased aggressiveness and shorter survival by modulating the expression and splicing events of key oncogenes such as FGFR4. Therefore, the inhibition of EIF4A3 could represent a novel therapeutic strategy to be used alone or combined with existing systemic or ablative therapies against HCC.

## CONFLICT OF INTEREST

The authors have no conflicts of interest to declare.

## Supporting information

Supporting Information

Supporting Information

Supporting Information

## Data Availability

The data of this study are available in https://doi.org/10.6084/m9.figshare.16689061.v1.
