# Supplementary material for: Spliceosomal profiling identifies EIF4A3 as a novel oncogene in hepatocellular carcinoma acting through the modulation of FGFR4 splicing
Source: Clin Transl Med. 2022 Nov 23;12(11):e1102. doi: 10.1002/ctm2.1102 (PMC9684617; doi:10.1002/ctm2.1102)
Supplement: Supplementary file 1 — Supporting Information [file CTM2-12-e1102-s001.pptx]

## Slide 1
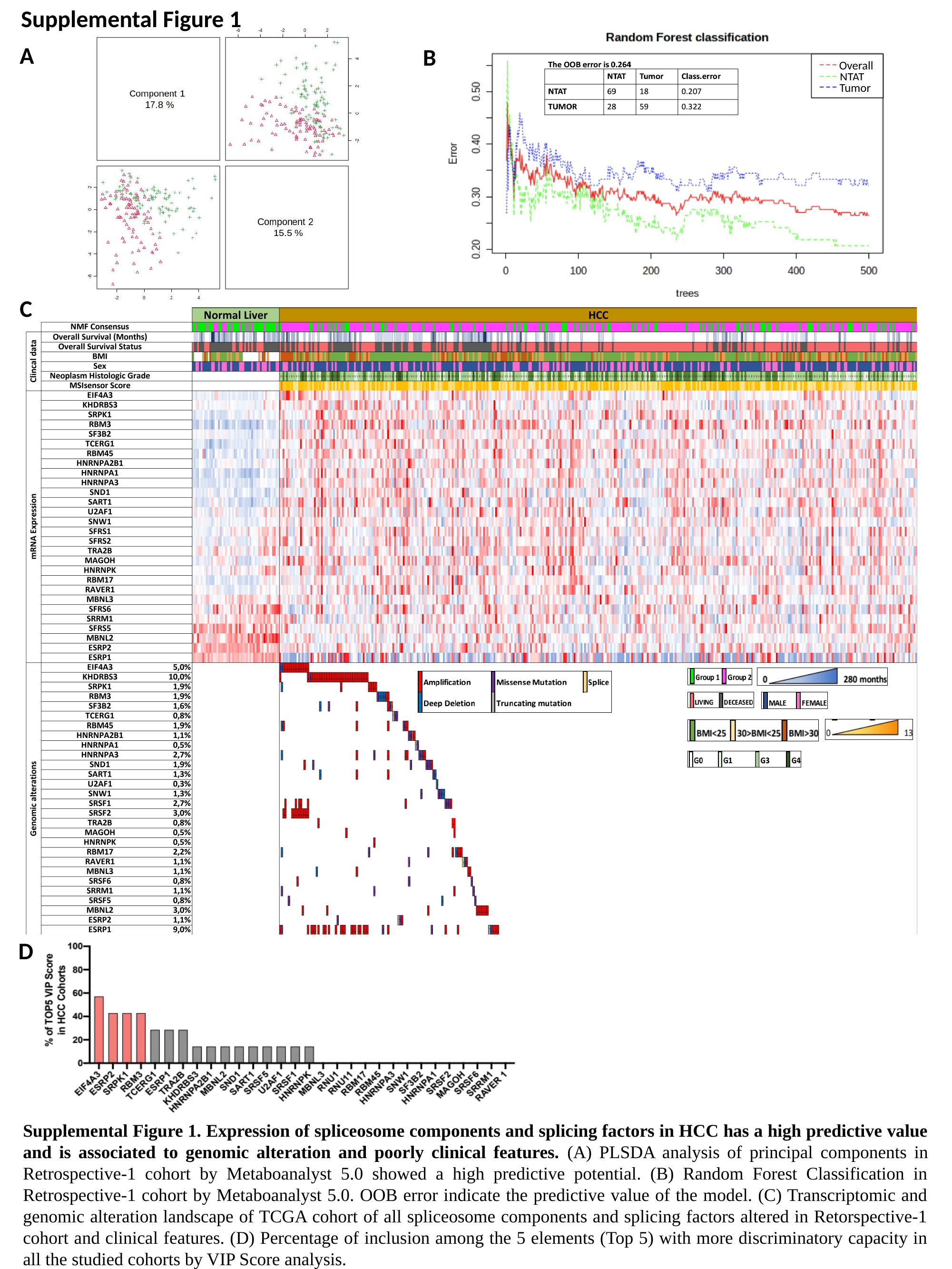

Supplemental Figure 1
Overall
NTAT
Tumor
A
B
C
D
Supplemental Figure 1. Expression of spliceosome components and splicing factors in HCC has a high predictive value and is associated to genomic alteration and poorly clinical features. (A) PLSDA analysis of principal components in Retrospective-1 cohort by Metaboanalyst 5.0 showed a high predictive potential. (B) Random Forest Classification in Retrospective-1 cohort by Metaboanalyst 5.0. OOB error indicate the predictive value of the model. (C) Transcriptomic and genomic alteration landscape of TCGA cohort of all spliceosome components and splicing factors altered in Retorspective-1 cohort and clinical features. (D) Percentage of inclusion among the 5 elements (Top 5) with more discriminatory capacity in all the studied cohorts by VIP Score analysis.

## Slide 2
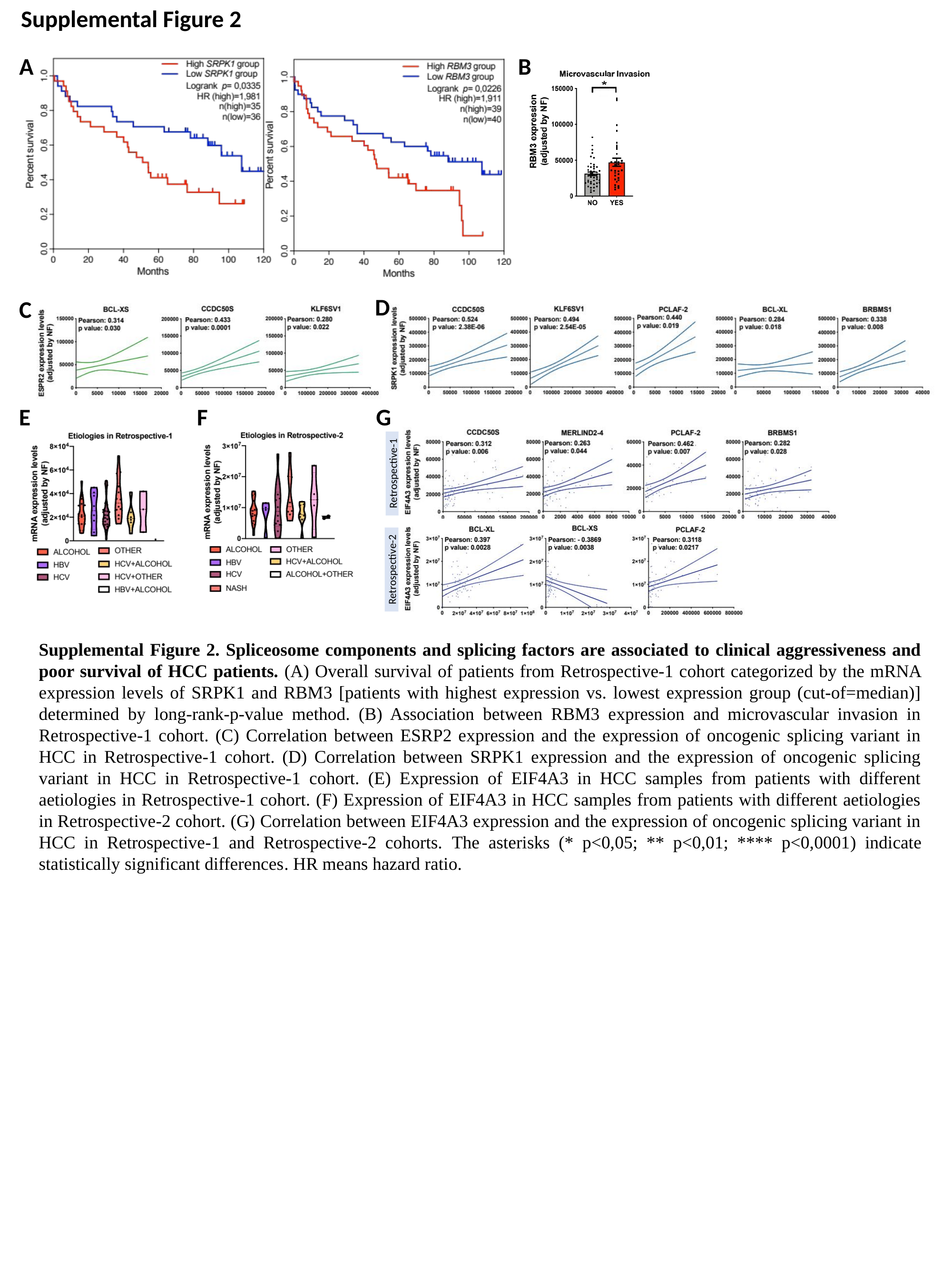

Supplemental Figure 2
B
A
D
C
F
G
E
Retrospective-1
Retrospective-2
Supplemental Figure 2. Spliceosome components and splicing factors are associated to clinical aggressiveness and poor survival of HCC patients. (A) Overall survival of patients from Retrospective-1 cohort categorized by the mRNA expression levels of SRPK1 and RBM3 [patients with highest expression vs. lowest expression group (cut-of=median)] determined by long-rank-p-value method. (B) Association between RBM3 expression and microvascular invasion in Retrospective-1 cohort. (C) Correlation between ESRP2 expression and the expression of oncogenic splicing variant in HCC in Retrospective-1 cohort. (D) Correlation between SRPK1 expression and the expression of oncogenic splicing variant in HCC in Retrospective-1 cohort. (E) Expression of EIF4A3 in HCC samples from patients with different aetiologies in Retrospective-1 cohort. (F) Expression of EIF4A3 in HCC samples from patients with different aetiologies in Retrospective-2 cohort. (G) Correlation between EIF4A3 expression and the expression of oncogenic splicing variant in HCC in Retrospective-1 and Retrospective-2 cohorts. The asterisks (* p<0,05; ** p<0,01; **** p<0,0001) indicate statistically significant differences. HR means hazard ratio.

## Slide 3
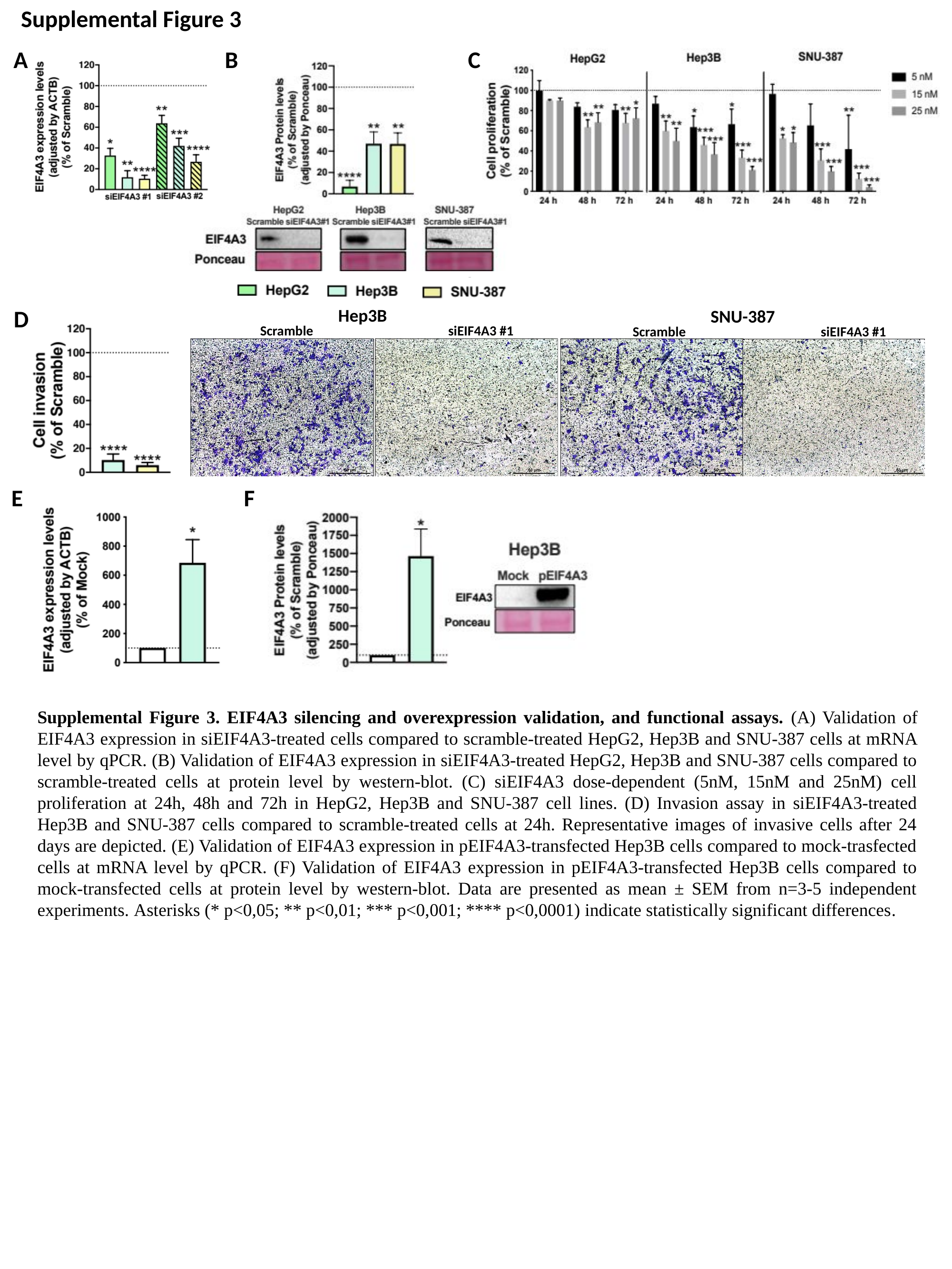

Supplemental Figure 3
A
B
C
D
Hep3B
SNU-387
Scramble
siEIF4A3 #1
Scramble
siEIF4A3 #1
E
F
Supplemental Figure 3. EIF4A3 silencing and overexpression validation, and functional assays. (A) Validation of EIF4A3 expression in siEIF4A3-treated cells compared to scramble-treated HepG2, Hep3B and SNU-387 cells at mRNA level by qPCR. (B) Validation of EIF4A3 expression in siEIF4A3-treated HepG2, Hep3B and SNU-387 cells compared to scramble-treated cells at protein level by western-blot. (C) siEIF4A3 dose-dependent (5nM, 15nM and 25nM) cell proliferation at 24h, 48h and 72h in HepG2, Hep3B and SNU-387 cell lines. (D) Invasion assay in siEIF4A3-treated Hep3B and SNU-387 cells compared to scramble-treated cells at 24h. Representative images of invasive cells after 24 days are depicted. (E) Validation of EIF4A3 expression in pEIF4A3-transfected Hep3B cells compared to mock-trasfected cells at mRNA level by qPCR. (F) Validation of EIF4A3 expression in pEIF4A3-transfected Hep3B cells compared to mock-transfected cells at protein level by western-blot. Data are presented as mean ± SEM from n=3-5 independent experiments. Asterisks (* p<0,05; ** p<0,01; *** p<0,001; **** p<0,0001) indicate statistically significant differences.

## Slide 4
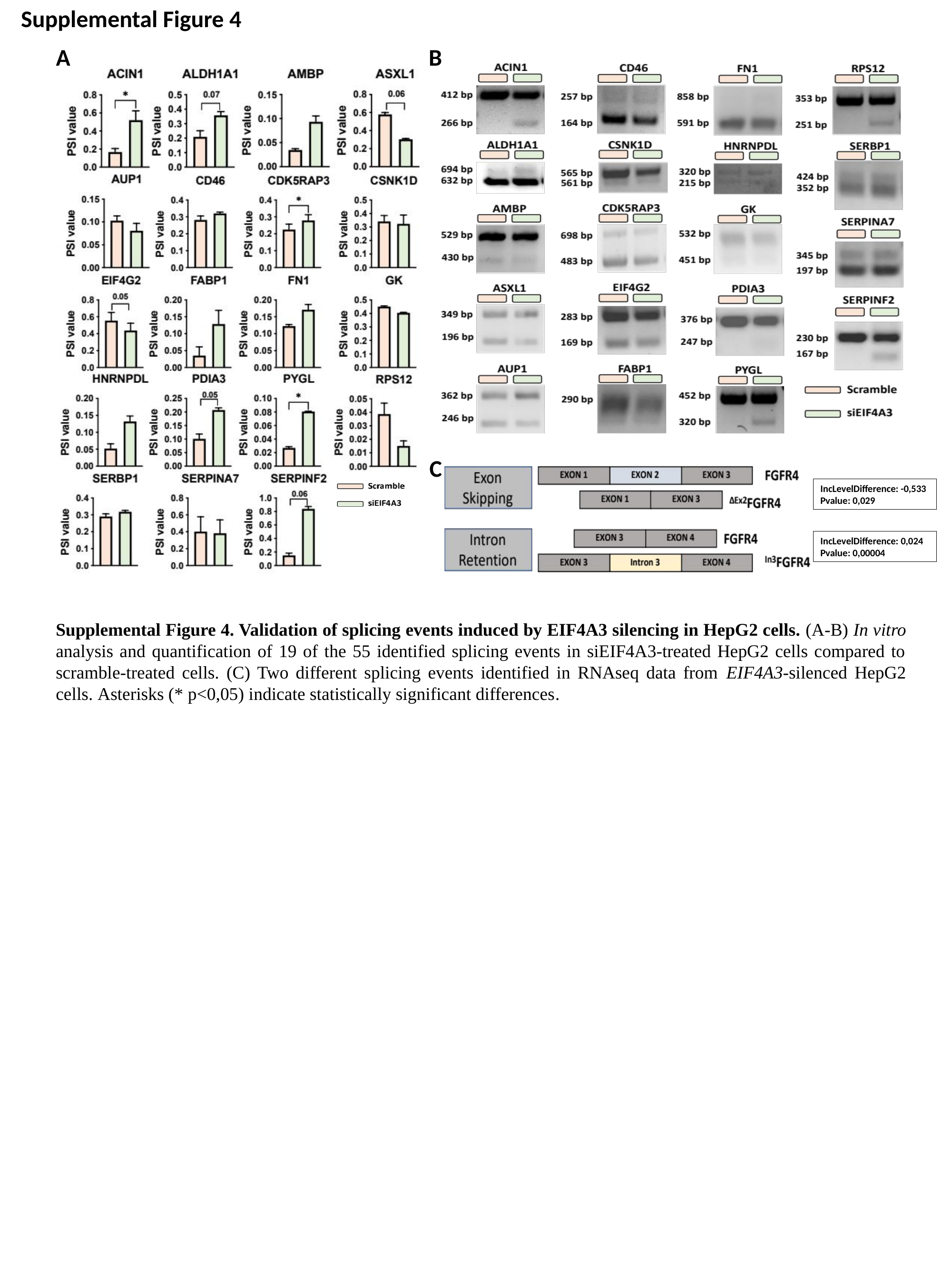

Supplemental Figure 4
B
A
C
IncLevelDifference: -0,533
Pvalue: 0,029
IncLevelDifference: 0,024
Pvalue: 0,00004
Supplemental Figure 4. Validation of splicing events induced by EIF4A3 silencing in HepG2 cells. (A-B) In vitro analysis and quantification of 19 of the 55 identified splicing events in siEIF4A3-treated HepG2 cells compared to scramble-treated cells. (C) Two different splicing events identified in RNAseq data from EIF4A3-silenced HepG2 cells. Asterisks (* p<0,05) indicate statistically significant differences.

## Slide 5
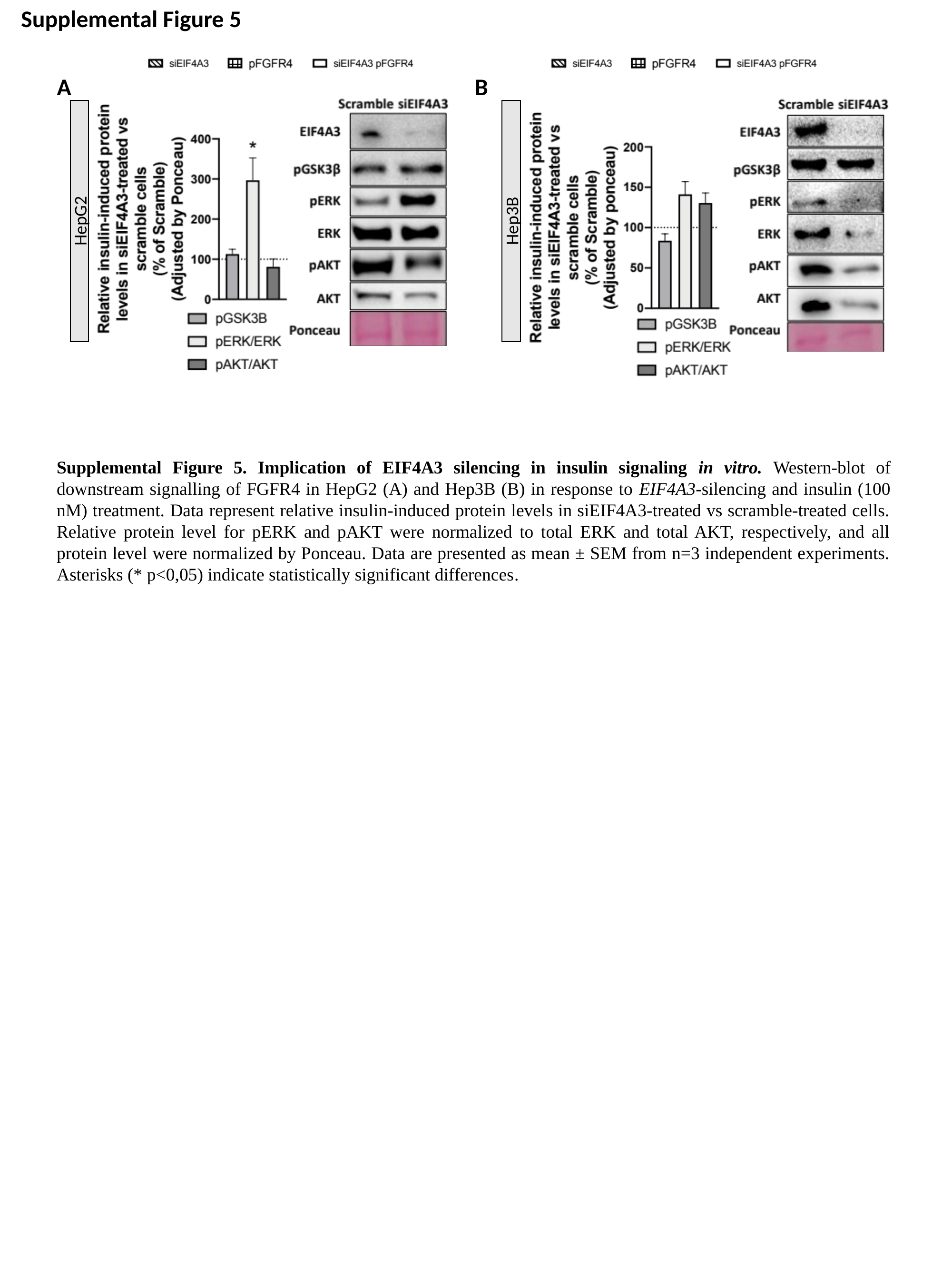

Supplemental Figure 5
A
B
HepG2
Hep3B
Supplemental Figure 5. Implication of EIF4A3 silencing in insulin signaling in vitro. Western-blot of downstream signalling of FGFR4 in HepG2 (A) and Hep3B (B) in response to EIF4A3-silencing and insulin (100 nM) treatment. Data represent relative insulin-induced protein levels in siEIF4A3-treated vs scramble-treated cells. Relative protein level for pERK and pAKT were normalized to total ERK and total AKT, respectively, and all protein level were normalized by Ponceau. Data are presented as mean ± SEM from n=3 independent experiments. Asterisks (* p<0,05) indicate statistically significant differences.
