# Supplementary material for: Spliceosomal profiling identifies EIF4A3 as a novel oncogene in hepatocellular carcinoma acting through the modulation of FGFR4 splicing
Source: Clin Transl Med. 2022 Nov 23;12(11):e1102. doi: 10.1002/ctm2.1102 (PMC9684617; doi:10.1002/ctm2.1102)
Supplement: Supplementary file 2 — Supporting Information [file CTM2-12-e1102-s003.docx]

**Supplemental Table 1**.

|  | **Retrospective-1** | **Retrospective-2** |
| --- | --- | --- |
| **Patients [n]** | **86** | **57** |
| **Age, y [median (IQR)]** | **60.6 (64-67)** | **61.2 (55-67.25)** |
| **Etiology [n (%)]** |  |  |
| - **HCV** | **30 (36.1)** | **17 (25.8)** |
| - **Alcohol** | **21 (25.3)** | **17 (25.8)** |
| - **HBV** | **11 (13.3)** | **5 (7.6)** |
| - **Other** | **5 (6)** | **8 (12.1)** |
| - **HCV + Alcohol** | **9 (10.8)** | **10 (15.2)** |
| - **HBV + Alcohol** | **1 (1.2)** | **1 (1.5)** |
| - **HCV + other** | **3 (3.6)** | **1 (1.5)** |
| - **Alcohol + other** | **0(-)** | **2(3)** |
| **Histological differentiation [n (%)]** |  |  |
| - **Well differentiated** | **30 (35.3)** | **33 (51.6)** |
| - **Moderately differentiated** | **50 (58.8)** | **26 (40.6)** |
| - **Poorly differentiated** | **5 (5.9)** | **5 (7.8)** |
| **Portal Hypertension [n (%)]** | **44 (5.2)** | **39 (59.1)** |
| **Microvascular invasion [n (%)]** | **33 (39.8)** | **23 (35.4)** |
| **Treated before surgery [n (%)]** | **23 (26.4)** | **38 (57.6)** |
| **Recurrence [n (%)]** | **39 (47)** | **20 (30.3)** |
| **Death [n (%)]** | **50 (61)** | **16 (24.2)** |

**Supplemental Table 1**. Demographic and clinical parameters of HCC patients included in the retrospective cohorts.

**Supplemental Table 2**.

|  | **HCC** | | **Cirrhosis** | **NAFLD** | **Control** |
| --- | --- | --- | --- | --- | --- |
| **Patients [n]** | **16** | | **25** | **28** | **21** |
| **Sex [n (%)]** | |  |  |  |  |
| - **Men** | **13 (81.3)** | | **15 (60.0)** | **21 (75.0)** | **15 (71.4)** |
| - **Women** | **3 (18.7)** | | **10 (40.0)** | **7 (25.0)** | **6 (28.6)** |

**Supplemental Table 2**. Clinical parameters of HCC, cirrhosis, NAFLD and control individuals included in the Liquid Biopsy Cohort (Prospective-1).

**Supplemental Table 3**.

|  | Transcript | Application | Forward | Reverse | Size (bp) |
| --- | --- | --- | --- | --- | --- |
| Spliceosome Components | *PRPF40A* | qPCR | GCTCGGAAGATGAAACGAAA | TGTCCTCAAATGCTGGCTCT | 130 |
|  | *RBM22* | qPCR | CTCTGGGTTCCAACACCTACA | GGCACAGATTTTGCATTCCT | 137 |
|  | *RNU1* | qPCR | ATCACGAAGGTGGTTTTCC | GCAGTCGAGTTTCCCACA | 114 |
|  | *RNU11* | qPCR | AAGGGCTTCTGTCGTGAGTG | CCAGCTGCCCAAATACCA | 108 |
|  | *RNU12* | qPCR | ATAACGATTCGGGGTGACG | CAGGCATCCCGCAAAGTA | 106 |
|  | *RNU2* | qPCR | CTCGGCCTTTTGGCTAAGAT | TATTCCATCTCCCTGCTCCA | 116 |
|  | *RNU4* | qPCR | TCGTAGCCAATGAGGTCTATCC | AAAATTGCCAGTGCCGACTA | 103 |
|  | *RNU4ATAC* | qPCR | GTTGCGCTACTGTCCAATGA | CAAAAATTGCACCAAAATAA | 85 |
|  | *RNU6* | qPCR | CGCTTCGGCAGCACATATA | AAAATATGGAACGCTTCACGAA | 101 |
|  | *RNU6ATAC* | qPCR | TGAAAGGAGAGAAGGTTAGCACTC | CGATGGTTAGATGCCACGA | 112 |
|  | *SNRNP200/RNU5* | qPCR | GGTGCTGTCCCTTGTTGG | CTTTCTTCGCTTGGCTCTTCT | 103 |
|  | *TCERG1* | qPCR | GAGGAGCCCAAAGAAGAGGA | CACCAGTCCAAACGACACAC | 112 |
|  | *U2AF1* | qPCR | GAAGTATGGGGAAGTAGAGGAGATG | TTCAAGTCAATCACAGCCTTTTC | 120 |
|  | *U2AF2* | qPCR | CTTTGACCAGAGGCGCTAAA | TACTGCATTGGGGTGATGTG | 130 |
| Splicing Factors | *CELF1* | qPCR | AACAGAAGAGAATGGCCCAGC | TGCTGAAGGAGTGCTAAATACTG | 121 |
|  | *CELF4* | qPCR | CCCCAGCAGCAGAGAGAA | GAAGCCGAAAGGGAGGAA | 108 |
|  | *DHX8* | qPCR | AGCCTGAGCATGAAGGATGT | AAGGGACAAGTGAGTGGGTCT | 132 |
|  | *DHX9* | qPCR | ACAGGTTCCCCAGTTCATTCT | TTTTTCCAGGCTCTTCTCCTC | 146 |
|  | *EIF4A3* | qPCR | TGACCTCTACGACACACTGACC | AAGTTGGCTTCCCTCATTTTC | 99 |
|  | *ELAVL4* | qPCR | AGCAAAACCAACCTCATCGT | TCCATACCCTAAACTCTGTCCTGT | 141 |
|  | *ESRP1* | qPCR | TTTTGGGATCACTGCTGGGG | TGTCCCACCTTCTTGTTGGC | 108 |
|  | *ESRP2* | qPCR | AGAGCCCAGCAGTCAATTGTT | GTCTCACTGTCCACCACATCAG | 96 |
|  | *HNRNPA1* | qPCR | AAAGCCCTGTCAAAGCAAGA | AGTTGTCATTCCCACCGAAA | 112 |
|  | *HNRNPA2B1* | qPCR | CAGAGTTCTAGGAGTGGAAGAGGA | CCATTATAGCCATCCCCAAA | 149 |
|  | *HNRNPA3* | qPCR | ATGGGGCACACTCACAGATT | GCATCCACCTCTTCAACACA | 102 |
|  | *HNRNPF* | qPCR | AGTCCCACAGAACCGAGATG | CCAACCCTGAGAAGAACTGAAC | 144 |
|  | *HNRNPG* | qPCR | AGAGATTATGCACCACCACCA | CACGATCACGACCATATCCA | 118 |
|  | *HNRNPK* | qPCR | CTGGGGTGTCAGTTGTTGG | TGGTTTCAGTGTTAGGGAAGG | 141 |
|  | *KHDRBS1* | qPCR | GAGCGAGTGCTGATACCTGTC | CACCAGTCTCTTCCTGCAGTC | 106 |
|  | *KHDRBS3* | qPCR | TGGTGCTGATTACTATGATTACGG | CTTTGCTGTCCTCGCTGAA | 115 |
|  | *MAGOH* | qPCR | GCCAACAACAGCAATTACAAGA | TTATTCTCTTCAGTTCCTCCATCAC | 88 |
|  | *MBNL1* | qPCR | TGACACCAATGACAACACAGTC | ATGTGCAGGGGGATGAAAG | 94 |
|  | *MBNL2* | qPCR | ACCACGCCTGTTATTGTTCC | TCCCTGCATACCTCCAGTTT | 101 |
|  | *MBNL3* | qPCR | TTATGCTCCAAAACGCTCAA | CCCAGGATGTGGTATGTAAGG | 107 |
|  | *NOVA1* | qPCR | TACCCAGGTACTACTGAGCGAG | CTGGTTCTGTCTTGGCCACAT | 124 |
|  | *PRPF19* | qPCR | CCAAGTTCCCAACCAAGTGT | GGCACAGTCTTCCCTCTCTTC | 145 |
|  | *PTBP1* | qPCR | TGGGTCGGTTCCTGCTATT | CAGATCCCCGCTTTGTAC | 111 |
|  | *RAVER1* | qPCR | GTAACCGCCGCAAGATACTG | CGAAGGCTGTCCCTTTGTATT | 126 |
|  | *RAVER2* | qPCR | TGGGAGAACCACCAAAAGAA | GCAGGGGATAAGCACAC | 88 |
|  | *RBM10* | qPCR | CAGCACTCCCTCAACATCCT | AGCACTTCTCTCGGCGTTT | 127 |
|  | *RBM17* | qPCR | CAAAGAGCCAAAGGACGAAA | TACATGCGGTGGAGTGTCC | 107 |
|  | *RBM25* | qPCR | GCTAAATGCCCCCTCACAG | CTGGAAATCTGCGGAAAATG | 86 |
|  | *RBM3* | qPCR | AAGCTCTTCGTGGGAGGG | TTGACAACGACCACCTCAGA | 98 |
|  | *RBM39* | qPCR | AGTTGGATGGGATACCGAGA | TTGCCCTGAGCTGAATTTTT | 102 |
|  | *RBM4* | qPCR | GTCCCACCTGCACCAATAAG | CCGCTCCATGTGTACGAAG | 104 |
|  | *RBM45* | qPCR | CCCATCAAGGTTTTCATTGC | TTCCCGCAGATCTTCTTCTG | 123 |
|  | *RBM5* | qPCR | TCAGGCACCAGCAACTCTC | CGGTCTCGGTATTTCATCTCTC | 124 |
|  | *RBM6* | qPCR | CCAGGATGGAGAGAGCAAAA | CAGTAGTAAGGCGGACATAGGG | 104 |
|  | *SART1* | qPCR | GGGCAGAGAAAAATGTGGAG | TGGACAGGATAGAGCGAGGT | 111 |
|  | *SF3B2* | qPCR | CTGCCAAACAGAAGCAAAAA | TGTGAGGGGACCTAAAACTTG | 97 |
|  | *SFPQ* | qPCR | TGGTAGGGGGTGAAAGTG | TTAAAAACAAGAAATGGGGAAATG | 125 |
|  | *SLU7* | qPCR | TGACCAGAGAGGACTGGAGAA | GAGGAATATGGGGGTTGATG | 111 |
|  | *SND1* | qPCR | ACTACGGCAACAGAGAGGTCC | GAAGGCATACTCCGTGGCT | 101 |
|  | *SNW1* | qPCR | ATGCGTGCCCAAGTAGAGAG | TCCCCATCCTCTTTTTCCA | 134 |
|  | *SRPK1* | qPCR | GAGCAAGAACATAACGGACCA | ACCCAACAAGCATTTCCAAG | 134 |
|  | *SRRM1* | qPCR | GTAGCCCAAGAAGACGCAAA | TGGTTCTGTGACGGGGAG | 108 |
|  | *SRRM4* | qPCR | CCTTCACCACCTCCTCAC | TTCGGCACATTCCAGACA | 113 |
|  | *SRSF1* | qPCR | TGTCTCTGGACTGCCTCCA | TGCCATCTCGGTAAACATCA | 98 |
|  | *SRSF10* | qPCR | CTACACTCGCCGTCCAAGAG | CCGTCCACAAATCCACTTTC | 103 |
|  | *SRSF2* | qPCR | TGTCCAAGAGGGAATCCAAA | GTTTACACTGCTTGCCGATACA | 113 |
|  | *SRSF3* | qPCR | TAACCCTAGATCTCGAAATGCATC | CATAGTAGCCAAAAGCCCGTT | 117 |
|  | *SRSF4* | qPCR | GGAACTGAAGTCAATGGGAGAA | CTTCGAGAGCGAGACCTTGA | 110 |
|  | *SRSF5* | qPCR | GCAAAAGGCACAGTAGGTCAA | TTTGCGACTACGGGAACG | 92 |
|  | *SRSF6* | qPCR | AGACCTCAAAAATGGGTACGG | CTTGCCGTTCAGCTCGTAA | 82 |
|  | *SRSF9* | qPCR | CCCTGCGTAAACTGGATGAC | AGCTGGTGCTTCTCTCAGGA | 87 |
|  | *TIA1* | qPCR | TAAATCCCGTGCAACAGCAGA | TATGCAGGAACTTGCCAACCA | 124 |
|  | *TRA2A* | qPCR | TCAAAGGAGGCTATGGAAAGG | TGTGTGCGCTCTCTTGGTTA | 90 |
|  | *TRA2B* | qPCR | GATGATGCCAAGGAAGCTAAAG | AGGTAGGTCTCCCCATGTAAATTC | 130 |
| SVs | *PCLAF-2* | qPCR | ATCGAGGAAAGAGCATGTCC | CAGGGTAAACAAGGAGACGTTA | 101 |
|  | *BRBMS1* | qPCR | GCTCTGAAGCCCATACATCG | GCCTTTTTGATGGCTGTCC | 74 |
|  | *MERLIND2-4* | qPCR | GGAGTTCAATTGCGAGTATGG | TTGGAAGCAATTCCTCTTGG | 80 |
|  | *^DEX2^FGFR4* | qPCR | AGGTGAGGAGGAGCCAGAG | TGCCCTCCTTGTACCAGTG | 137 |
|  | *KLF6-SV1* | qPCR | CCTCGCCAGGGAAGGAGAA | CGGTGTGCTTTCGGAAGTG | 80 |
|  | *BCL-XL* | qPCR | GATGGCCACTTACCTGAATGA | TGCTGCATTGTTCCCATAGA | 94 |
|  | *BCL-XS* | qPCR | GAGCTTTGAACAGGATACTTTTGTG | GAAGAGTGAGCCCAGCAGAA | 97 |
|  | *CCDC50S* | qPCR | GCTGGCTATTGAGGCAGAG | TGGCTTCATTCCTCCATCTT | 178 |
| HK | *ACTB* | qPCR | ACTCTTCCAGCCTTCCTTCCT | CAGTGATCTCCTTCTGCATCCT | 176 |
|  | *GAPDH* | qPCR | AATCCCATCACCATCTTCCA | AAATGAGCCCCAGCCTTC | 122 |
|  | *HPRT* | qPCR | CTGAGGATTTGGAAAGGGTGT | TAATCCAGCAGGTCAGCAAAG | 157 |

**Supplemental Table 3**. Specific primers for human transcripts used in this study, including spliceosome components, associated splicing factors, three housekeeping genes (HK) and splicing varians (SVs)that were specifically designed and used in qPCR-based microfluidic assays, RT-qPCR and PCR. Official name of the genes, primers application, primers sequences and product sizes of the amplification products are included.

**Supplemental Table 4**.

| Transcript | Application | Forward | Reverse | Size (bp) |
| --- | --- | --- | --- | --- |
| *FGFR4* | PCR | GCTGGAGCTGGGAGTGAG | TCAGGTAGGAAGCTGGCAAT | 383 |
| *ACIN1* | PCR | TCCCCAGGTGTCAGTAGAGG | CTCGGCATAGTCAGCACAAA | 412 |
| *ALDH1A1* | PCR | GAAGAAAGAAGGGGCCAAAC | ACTTGGGGGTCACATTTCAG | 632 |
| *AMBP* | PCR | TGCTACCCCAAGAAGAGGAA | TTTATTTGGACCCAGGTTGC | 529 |
| *ASXL1* | PCR | AAAAGACTGGGGTGATGCTG | TGTGATGGTAAGGCATGGAA | 349 |
| *AUP1* | PCR | CTGATGTGCAACTGGCAACT | TTGAGCTCAGTCAGCCTCCT | 362 |
| *CD46* | PCR | TGGGTCATTGCTGTGATTGT | CCTCTCTGCTCTGCTGGAGT | 257 |
| *CSNK1D* | PCR | CATGGAGAGAGAGCGGAAAG | CAGTGGAATCGTCAGGGAGT | 501 |
| *CDK5RAP3* | PCR | CCTGGTTCGGAATGTCAACT | GACACTGCCTCTACCCCAAA | 698 |
| *EIF4G2* | PCR | ATCGCAGTTTGGAGAGATGG | CTGTCCCAGAGGTGGTGTTT | 283 |
| *FABP1* | PCR | GCAGAGCCAGGAAAACTTTG | CGTTGAGTTCGGTCACAGAC | 290 |
| *FN1* | PCR | ATTGATGCACCATCCAACCT | AACAACCTCTTCCCGAACCT | 858 |
| *HNRNPDL* | PCR | GTGGCTATGGCGGATATGAT | TCTTCAATGTCGTCCTGCAA | 215 |
| *GK* | PCR | AGTCTCGAACCCGAGGATTT | GGGAATGGAGCAGGATGTTA | 451 |
| *PDIA3* | PCR | CTGATTTTGGCTTGGAGAGC | GTGGCATCCATCTTGGCTAT | 376 |
| *PYGL* | PCR | GACTTGGCAATGGTGGTCTT | ATAGAGGACCCGGGAGATGT | 452 |
| *RPS12* | PCR | TTGCTGCTGGAGGTGTAATG | GACATCCTTGGCCTGAGACT | 353 |
| *SERBP1* | PCR | CTATTCGAGGTCGTGGTGGT | GCCCGGTCCTTATTTTGAAT | 424 |
| *SERPINA7* | PCR | AGCAAGAATGCTCTGGCACT | AGGCTGATCCGAAAGTTCAA | 345 |
| *SERPINF2* | PCR | GTTGTGTGTGGCAGCAAGG | GACTCTTCAGGGCAGTCTGG | 230 |

**Supplemental Table 4**. Specific primers for human transcripts used in this study, designed and used in PCR to identify specific Splicing events. Official name of the genes, primers application, primers sequences and product sizes of the amplification products are included.
