# Supplementary material for: Spliceosomal profiling identifies EIF4A3 as a novel oncogene in hepatocellular carcinoma acting through the modulation of FGFR4 splicing
Source: Clin Transl Med. 2022 Nov 23;12(11):e1102. doi: 10.1002/ctm2.1102 (PMC9684617; doi:10.1002/ctm2.1102)
Supplement: Supplementary file 3 — Supporting Information [file CTM2-12-e1102-s002.docx]

**Supplemental Material and Methods**

***In silico* analysis of HCC cohorts**

Gene Expression Profiling Interactive Analysis (GEPIA), an interactive web server for analysing the RNA sequencing expression data from The Cancer Genome Atlas Program [1] and the Genotype-Tissue Expression (GTEx) projects, was used to analyse the expression level of all spliceosome components and splicing factors included in the study in tumor and normal tissues, and the survival of HCC patients in TCGA cohort.

To analyse the expression levels of all spliceosome components and splicing factors included herein in other validation cohorts, the Oncomine database was used, which includes data from different HCC cohorts: Wurmbach Liver (10 normal liver vs. 35 HCC)[2], Mas liver (19 normal liver vs. 38 HCC)[3], Roessler Liver (21 normal liver vs. 22 HCC) and Roessler Liver 2 (220 normal liver vs. 225 HCC)[4]. Cancer Institute Clinical Proteomic Tumor Analysis Consortium (CPTAC) portal was used to obtain data from proteomic studies in paired tumor and adjacent liver tissues from 159 patients with HBV-related HCC[5].

**Bioinformatic analysis**

Metaboanalyst 5.0 was used to perform the majority of bioinformatic analysis. PLS-DA analysis is a statistical method similar to principal components analysis[6] that changes the maximum variance found by a linear regression model in a different dimension showing the best elements to discriminate between different experimental groups (NTAT and tumor). VIP score analysis is a measure of a variable's importance in the PLS-DA model. It summarizes the contribution a variable makes to the model. The VIP score of a variable is calculated as a weighted sum of the squared correlations between the PLS-DA components and the original variable. Metaboanalyst 5.0 allowed us to use the receiver operating characteristic (ROC) curve-based approach for identifying potential biomarkers and evaluating their performance. Classical univariate ROC curve analysis as well as multivariate ROC curve analysis based on PLS-DA, were performed.

Gene Set Enrichment Analysis (GSEA) was performed by Genepattern (<https://www.genepattern.org/>) in Reactome. GSEA analysis is a computational method that determines whether an a priori defined set of genes shows statistically significant, concordant differences between two biological states (e.g. phenotypes).

STRING analysis (<https://www.string-db.org/>) was performed for Differential Expressed and Splicing quantified genes (FDR>0.05). STRING allow us to know protein-protein interaction and clustering the genes in biological function, cellular components, etc.

**RNAseq data analysis**

RNAseq data from EIF4A3-shRNA knockdown HepG2 cells was available through ENCODE Portal database [7, 8]. Differential expression quantifications (ENCFF236CND) were analyzed by sva, tximport, deseq2, salmon and cqn software and mapping assembly in hg19 genome. In addition, differentially splicing quantifications (ENCFF983CUA) were analyzed, by rmats and sva software and mapping assembly in hg19 genome, in RNAseq to identify key splicing events when EIF4A3 was silenced or not in HepG2 cell line.

**Cell lines and treatments**

Liver cancer cell lines HepG2 (hepatoblastoma), Hep3B (HCC) and SNU-387 (HB-8065, HCC) were used (ATCC, Manassas, USA). HepG2 and Hep3B were cultured in Minimum Essential Media (Thermo Fisher, Madrid, Spain), with 10% fetal bovine serum (FBS, Sigma-Aldrich, Madrid, Spain), 0.2% antibiotic-antifungal (Gentamicin/amphotericin-B, Thermo Fisher) and 0.5% sodium pyruvate [9]. SNU-387 cells were cultured in Roswell Park Memorial Institute medium (RPMI-1640, Thermo Fisher), with 10% FBS, 0.2% antibiotic-antifungal and 0.5% Glutamine (Thermo Fisher)[9]. Cells were maintained at 37°C and 5% CO2, under sterile conditions, periodically validated by short tandem repeat analysis (GenePrint 10 System, Promega, Barcelona, Spain) and tested for mycoplasma contamination [9-11].

**RNA isolation and retrotranscription**

Total RNA from frozen tissues was isolated using the AllPrep DNA/RNA/Protein Kit (Qiagen, Madrid, Spain), total RNA from FFPE tissues was isolated using the Maxwell FFPE Purification Kit (Promega) and total RNA from cell lines was isolated using TRI Reagent (Sigma-Aldrich). RNA extraction, quantification and reverse transcription were performed following previously reported protocols [9]. RNA extraction was followed by DNase treatment (Promega, Barcelona, Spain). The amount and purity of RNA recovered were determined using the NanoDrop 2000 spectrophotometer (Thermo Fisher). Finally, RNA (1μg) was reverse transcribed to cDNA using the RevertAid First-Strand cDNA Synthesis Kit (Thermo Fisher).

**RNA expression analysis by conventional qPCR**

RNA expression levels of the transcripts of interest were determined by conventional qPCR in cell lines and xenografted tumors. Specific primers for human transcripts (Supplemental Table 3) were specifically designed with Primer3 software (Applied Biosystems, Foster City, CA). Conventional qPCR reactions were carried out using the Stratagene Mx3000p system with the Brilliant III SYBR Green Master Mix (Stratagene, La Jolla, CA)[9]. The expression level of each transcript was adjusted by the expression of ACTB. In all cases, these housekeeping genes exhibited a stable expression among experimental groups.

**Semi-quantitative PCR**

To determine the presence of splicing events of genes identified with differential expression and splicing quantifications in liver cancer cell lines, semi-quantitative PCR was implemented by using specific primers designed with Primer3 (Supplemental Table 4) in the T100^TM^ Thermal Cycler (Bio-Rad, Madrid, Spain). Specifically, 50 ng of cDNA were denatured at 95ºC for 3 minutes, followed by 45 cycles of 95ºC for 30 seconds, 57ºC for 30 seconds, 72ºC for 30 seconds, and a final extension at 72ºC for 5 minutes. The PCR products were analyzed by electrophoresis in 2% agarose gel, and the results were documented by the Molecular Imager Gel Doc™ XR-Universal H (Bio-Rad, Madrid, Spain).

**Quantitative real-time PCR (qPCR) dynamic array based on microfluidic technology**

A qPCR dynamic array based on microfluidic technology that allows the simultaneous determination of the expression of 70 transcripts in 96 samples (Fluidigm, San Francisco, CA, USA) was used. These 70 splicing-related elements were selected based on different criteria: 1) Importance in the splicing process; 2) Relevance in the function of the spliceosome; 3) Relationship with splicing variants found in HCC; 4) Implication in other tumor pathologies. Specific primers for human transcripts including components of the major spliceosome (n=10), minor spliceosome (n=4), associated splicing factors (n=56) and three housekeeping genes (*ACTB*, *GAPDH* and *HPRT*) were designed with the Primer3 software (Applied Biosystems, Foster City, CA, USA) (Supplemental Table 3). Preamplification, exonuclease treatment and qPCR dynamic array based on microfluidic technology were implemented as recently reported [11, 12]. To control for variations in the efficiency of the retro-transcription reaction, mRNA copy numbers of the different transcripts analysed were adjusted by normalization factor, calculated with the expression levels of *ACTB* and *GAPDH* (the most stably expressed housekeeping genes among groups) using GeNorm 3.3 [13].

**Western blotting**

Liver cancer cell lines were processed to analyze protein levels by western blot after 24 h of EIF4A3 siRNA transfection, as previously described[9]. Briefly, 150,000 cells were seeded in 6-well plates. When cells were silenced by siEIF4A3, cells were treated with FGF19 (50 nM) (Recombinant Human FGF-19 Protein, 969-FG, R&D) and Insulin (100nM) (91077C-100MG, Sigma). Proteins were extracted using pre-warmed Sodium Dodecyl Sulfate-Dithiothreitol (SDS-DTT) buffer (62.5 mM Tris-HCl, 2% SDS, 20% glycerol, 100 mM DTT, and 0.005% bromophenol blue). Then, proteins were sonicated for 10 s and boiled for 5 min at 95 °C. Proteins were separated by SDS-PAGE and transferred to nitrocellulose membranes (Millipore, Billerica, MA, USA). Membranes were blocked with 5% non-fat dry milk in Tris-buffered saline/0.05% Tween-20 and incubated overnight with the specific primary antibodies for EIF4A3 (Sigma, HPA021878), phospho-AKT(Ser473) (#4060S, Cell Signaling, Barcelona, Spain), AKT (#9272S; Cell Signaling), phospho-ERK (Thr202/Tyr204) (#4370S, Cell Signaling), ERK (sc-154, Santa Cruz Biotechnology), Phospho-GSK-3-beta (Ser9) (D3A4) (#9322, Cell Signaling), phosphor-SRC (Y419)(ab185617, Abcam), SRC (701396, Thermofisher), as well as with the appropriate secondary antibodies: HRP-conjugated goat antirabbit IgG (#7074S, Cell Signaling). Proteins were detected using an enhanced chemiluminescence detection system (GEHealthcare, Madrid, Spain) with dyed molecular weight markers (Bio-Rad, Madrid, Spain). A densitometry analysis of the bands obtained was carried out with ImageJ software, using total protein levels by Ponceau, or total ERK and AKT levels as normalizing factors. All experiments were performed, at least, with three independent cell preparations.

**Measurements of cell proliferation**

Cell proliferation was determined by Resazurin Reagent (# CA035; Canvax Biotech, Córdoba, Spain) in

all cell lines[9]. Briefly, 10,000 cells were seeded on each well (96-wells plates) and serum-starved for 24h. Cell proliferation in response to silencing and overexpression was evaluated every 24h for 3 days. In all experiments, cells were seeded per quadruplicate.

**Measurement of cell migration capacity**

Cell migration was evaluated by wound healing assay. Briefly, silenced or overexpressed cells were plated in 24-96 wells plates in triplicates and, when confluent, serum starved for 1h[9]. A wound was made in the center of each well and the wound-healing capacity determined after 24h. Cells were cultured in serum-free medium to prevent proliferation. In all experiments, cells were seeded in quadruplicate and analysed by ImageJ (FIJI) software.

**Clonogenic assay**

For clonogenic assays, 2,000 silenced or overexpressed cells were seeded in 6-well plates and incubated for 10 days at 37°C and 5% CO2[9]. Finally, cells were stained with crystal violet solution (6% glutaraldehyde, 0.5% Violet Crystal) and the number of colonies formed (corresponding to accumulations of more than 50 cells) analyzed using ImageJ. In all cases, the experiments were performed in triplicate.

**Tumorspheres formation**

To determine the ability of the cell lines to form tumorospheres after the silencing or overexpression of gen of interest, 10,000 cells were seeded for SNU-387 and 5,000 cells for Hep3B and HepG2, in 24-well Corning Costar Ultra-Low Attachment multiwell plates (Sigma-Aldrich) [9]. Cells were seeded in medium with 10% FBS and supplemented with growth factors (FGF, EGF, B27), which were refreshed every 4 days. After 10 days, the number and size of the tumorospheres were analyzed and the mean area of the tumorospheres was determined using Image J. In all cases, the tests were performed in duplicate.

**Measurements of cell invasion**

Cell invasion was evaluated by a modified Boyden chamber method, using a 48-well chemotaxis chamber (NeuroProbe, Gaithersburg, MD) [9]. Briefly, 25000 Hep3B and 20000 SNU-387 cells per well were seeded onto an 8µM pore PDVF membrane (NeuroProbe) previously treated with collagen type IV (BD Biosciences, San Jose, CA). The lower well was filled with FBS-free media (negative control) or 10% FBS, including two replicates per condition. After 24h, non-migrated cells were removed and migrated cells fixed in crystal violet solution (6% glutaraldehyde, 0.5% Violet Crystal). Images from random fields were taken and analysed with ImageJ. The experiments were performed in triplicate.

**References**

1. Consortium ITP-CAoWG. Pan-cancer analysis of whole genomes. Nature. 2020;578(7793):82-93.

2. Wurmbach E, Chen YB, Khitrov G, Zhang W, Roayaie S, Schwartz M, et al. Genome-wide molecular profiles of HCV-induced dysplasia and hepatocellular carcinoma. Hepatology. 2007;45(4):938-47.

3. Mas VR, Maluf DG, Archer KJ, Yanek K, Kong X, Kulik L, et al. Genes involved in viral carcinogenesis and tumor initiation in hepatitis C virus-induced hepatocellular carcinoma. Mol Med. 2009;15(3-4):85-94.

4. Roessler S, Jia HL, Budhu A, Forgues M, Ye QH, Lee JS, et al. A unique metastasis gene signature enables prediction of tumor relapse in early-stage hepatocellular carcinoma patients. Cancer Res. 2010;70(24):10202-12.

5. Gao Q, Zhu H, Dong L, Shi W, Chen R, Song Z, et al. Integrated Proteogenomic Characterization of HBV-Related Hepatocellular Carcinoma. Cell. 2019;179(2):561-77 e22.

6. Ruiz-Perez D, Guan H, Madhivanan P, Mathee K, Narasimhan G. So you think you can PLS-DA? BMC Bioinformatics. 2020;21(Suppl 1):2.

7. Consortium EP. An integrated encyclopedia of DNA elements in the human genome. Nature. 2012;489(7414):57-74.

8. Davis CA, Hitz BC, Sloan CA, Chan ET, Davidson JM, Gabdank I, et al. The Encyclopedia of DNA elements (ENCODE): data portal update. Nucleic Acids Res. 2018;46(D1):D794-D801.

9. Lopez-Canovas JL, Del Rio-Moreno M, Garcia-Fernandez H, Jimenez-Vacas JM, Moreno-Montilla MT, Sanchez-Frias ME, et al. Splicing factor SF3B1 is overexpressed and implicated in the aggressiveness and survival of hepatocellular carcinoma. Cancer Lett. 2021;496:72-83.

10. Fuentes-Fayos AC, Vazquez-Borrego MC, Jimenez-Vacas JM, Bejarano L, Pedraza-Arevalo S, F LL, et al. Splicing machinery dysregulation drives glioblastoma development/aggressiveness: oncogenic role of SRSF3. Brain. 2020;143(11):3273-93.

11. Jimenez-Vacas JM, Herrero-Aguayo V, Montero-Hidalgo AJ, Gomez-Gomez E, Fuentes-Fayos AC, Leon-Gonzalez AJ, et al. Dysregulation of the splicing machinery is directly associated to aggressiveness of prostate cancer. EBioMedicine. 2020;51:102547.

12. Del Rio-Moreno M, Alors-Perez E, Gonzalez-Rubio S, Ferrin G, Reyes O, Rodriguez-Peralvarez M, et al. Dysregulation of the Splicing Machinery Is Associated to the Development of Nonalcoholic Fatty Liver Disease. J Clin Endocrinol Metab. 2019;104(8):3389-402.

13. Vandesompele J, De Preter K, Pattyn F, Poppe B, Van Roy N, De Paepe A, et al. Accurate normalization of real-time quantitative RT-PCR data by geometric averaging of multiple internal control genes. Genome Biol. 2002;3(7):RESEARCH0034.
